# A rare sequence variant in intron 1 of *THAP1* is associated with primary dystonia

**DOI:** 10.1002/mgg3.67

**Published:** 2014-02-11

**Authors:** Satya R Vemula, Jianfeng Xiao, Yu Zhao, Robert W Bastian, Joel S Perlmutter, Brad A Racette, Randal C Paniello, Zbigniew K Wszolek, Ryan J Uitti, Jay A Van Gerpen, Peter Hedera, Daniel D Truong, Andrew Blitzer, Monika Rudzińska, Dragana Momčilović, Hyder A Jinnah, Karen Frei, Ronald F Pfeiffer, Mark S LeDoux

**Affiliations:** 1 Departments of Neurology and Anatomy & Neurobiology, University of Tennessee Health Science CenterMemphis, Tennessee, 38163; 2 Bastian Voice InstituteDowners Grove, Illinois; 3 Department of Neurology, Washington University School of MedicineSt. Louis, Missouri; 4 Department of Otolaryngology-Head and Neck Surgery, Washington University School of MedicineSt. Louis, Missouri; 5 Department of Neurology, Mayo ClinicJacksonville, Florida, 32224; 6 Department of Neurology, Vanderbilt UniversityNashville, Tennessee; 7 Parkinson's & Movement Disorder InstituteFountain Valley, California, 92708; 8 New York Center for Voice and Swallowing DisordersNew York, New York; 9 Department of Neurology, Jagiellonian University Medical College in KrakowKraków, Poland; 10 Clinic for Child Neurology and Psychiatry, Medical Faculty University of BelgradeBelgrade, Serbia; 11 Departments of Neurology, Human Genetics, and Pediatrics, School of Medicine, Emory UniversityAtlanta, Georgia, 30322; 12 Department of Neurology, Loma Linda University Health SystemLoma Linda, California, 92354

**Keywords:** Dystonia, DYT6, intronic variant, minigene assay, THAP1

## Abstract

Although coding variants in *THAP1* have been causally associated with primary dystonia, the contribution of noncoding variants remains uncertain. Herein, we examine a previously identified Intron 1 variant (c.71+9C>A, rs200209986). Among 1672 subjects with mainly adult-onset primary dystonia, 12 harbored the variant in contrast to 1/1574 controls (*P* < 0.01). Dystonia classification included cervical dystonia (*N* = 3), laryngeal dystonia (adductor subtype, *N* = 3), jaw-opening oromandibular dystonia (*N* = 1), blepharospasm (*N* = 2), and unclassified (*N* = 3). Age of dystonia onset ranged from 25 to 69 years (mean = 54 years). In comparison to controls with no identified *THAP1* sequence variants, the c.71+9C>A variant was associated with an elevated ratio of Isoform 1 (NM_018105) to Isoform 2 (NM_199003) in leukocytes. In silico and minigene analyses indicated that c.71+9C>A alters *THAP1* splicing. Lymphoblastoid cells harboring the c.71+9C>A variant showed extensive apoptosis with relatively fewer cells in the G2 phase of the cell cycle. Differentially expressed genes from lymphoblastoid cells revealed that the c.71+9C>A variant exerts effects on DNA synthesis, cell growth and proliferation, cell survival, and cytotoxicity. In aggregate, these data indicate that *THAP1* c.71+9C>A is a risk factor for adult-onset primary dystonia.

## Introduction

Dystonia is a neurological disorder characterized by sustained or intermittent involuntary muscle contractions leading to abnormal postures and movements that may be tremulous (Fahn et al. 1998; Albanese et al. 2013). Physiologically, dystonia may be associated with atypical cocontractions of agonist and antagonist muscle groups, impaired control of somatotopically contiguous muscles, and reduced inhibition of spinal and brainstem reflexes (LeDoux 2012a). Dystonia is classified along two axes: clinical characteristics and etiological categories. Clinical characteristics include age of onset, body distribution, temporal pattern, coexistence of other movement disorders, and other neurological manifestations (Fahn et al. 1998; LeDoux 2012b; Albanese et al. 2013). Etiological categories include nervous system pathology and inherited. In general, adult-onset dystonia without evidence of overt degeneration or structural lesions of the nervous system is referred to as primary or isolated dystonia and inherited in autosomal-dominant fashion with reduced penetrance (Fahn et al. 1998; LeDoux 2012b). To date, coding mutations in *TOR1A*, *THAP1*, *CIZ1*, *GNAL*, and *ANO3* have been causally associated with primary dystonia (Ozelius et al. 1997; Fuchs et al. 2009, 2012; Xiao, et al., 2010; Xiao et al. 2012; Vemula et al. 2013). The role of large structural and noncoding variants in the etiopathogenesis of primary dystonia remains largely unexplored (Moscovich et al. 2013).

Among the known genetic causes of adult-onset primary dystonia, *THAP1* dystonia appears to be associated with the most diverse ages of onset and anatomical distributions (Fuchs et al. 2009; Xiao et al. 2010; LeDoux et al. 2012). *THAP1* encodes Thanatos-associated THAP domain containing, apoptosis-associated protein 1, encoding DNA-binding transcription factor THAP1. Located on Chr.8p11.21 (42,691,817–42,698,474: 6658 bp), the major isoform of this gene (NM_018105) contains three exons. THAP1 harbors a zinc-binding THAP domain, nuclear localization signal, and coiled-coiled domain. This zinc-binding domain is present in other proteins that are involved in cell-cycle control, apoptosis, and transcriptional regulation. THAP proteins are absent from plants and bacteria, but found in *Drosophila*, *Caenorhabditis elegans*, *Xenopus*, zebrafish, and mammals (Roussigne et al. 2003). Monomeric THAP1 shows low affinity for DNA (Campagne et al. 2010), and amino acid residues 154–166 are critical for dimerization (Sengel et al. 2011). THAP1 is widely expressed in brain and extraneural tissues including whole blood (Zhao et al. 2013).

THAP1 binds to specific DNA sequences, regulating cell proliferation and the G1/S checkpoint through pRB/E2F cell-cycle target genes (Clouaire et al. 2005; Cayrol et al. 2007; Sabogal et al. 2010; LeDoux et al. 2012, 2013). For instance, RRM1 is a direct transcriptional target of THAP1 (Cayrol et al. 2007). Both overexpression and knockdown of THAP1 in endothelial cells (ECs) inhibits proliferation.

To date, over 80 disease-specific sequence variants have been localized to the coding regions of *THAP1* and associated with focal, segmental, multifocal, and generalized dystonia with age of onset ranging from 3 to over 60 years (LeDoux et al. 2012). Consistent with the characteristically reduced penetrance of adult-onset dystonia, several asymptomatic carriers have been identified in the relatives of probands. The broad clinical heterogeneity of THAP1 dystonia and low penetrance of known coding variants suggest that noncoding variants in *THAP1* could contribute to the risk of developing adult-onset primary dystonia. In 2010, we identified a sequence variant (c.71+9C>A) (rs200209986) near the donor site of Intron 1 that might increase the risk of developing primary dystonia (Xiao, et al. 2010). The variant was found in 7/1210 subjects with dystonia but only 1/600 controls. Although the control did not manifest dystonia, she did report trismus and had experienced seizures as a child. Herein, we present the results of a larger definitive case–control study of *THAP1* c.71+9C>A in 1672 subjects with primary, mainly adult-onset, dystonia and 1574 neurologically normal Caucasian controls. Moreover, we characterize the relative effects of *THAP1* c.71+9C>A on cell-cycle control, splicing, and gene expression.

## Materials and Methods

### Human subjects

All human studies were conducted in accordance with the Declaration of Helsinki with formal approval from the institutional review boards at each participating study site. All subjects gave written informed consent. Recruitment of patients with primary dystonia and neurologically normal controls has been described (Xiao, et al. 2010; LeDoux et al. 2012; Vemula et al. 2013). Demographics and phenotypes for dystonia and control subjects are presented in Table 1 and Table S1. The control subject with trismus reported earlier (Xiao, et al. 2010) was excluded from the analyses reported herein because of uncertainty regarding how her phenotype should be classified. Of note, the data contained in Table 1 do not include the family members of probands. In addition, all neurologically normal controls and all subjects classified as primary dystonia were Caucasians of European descent. Minor allele frequencies (MAFs) were compared with those reported in 1000 Genomes (http://www.1000genomes.org) and Exome Variant Server (EVS) (http://evs.gs.washington.edu) databases using Fisher's exact test or Chi-square with Yates correction.

**Table 1:** Clinical diagnoses and demographics

			Gender		
Clinical diagnosis	Number (age of onset)^1^	Family history^2^	Male	Female	Number of subjects with c.71+9C>A
Laryngeal dystonia	472 (45.4 ± 15.7, 7–85)	8.3%	110	362	3
Cervical dystonia	509 (44.6 ± 13.6, 4–76)	10.0%	118	391	3
Blepharospasm	198 (58.1 ± 9.4, 20–73)	10.1%	61	137	2
Hand–forearm dystonia	52 (34.9 ± 13.0, 7–60)	9.6%	23	29	0
Oromandibular dystonia	18 (52.5 ± 12.3, 20–70)	11.1%	4	14	1
Other primary focal dystonia	38 (43.3 ± 18.1, 10–74)	13.2%	15	23	0
Segmental dystonia	143 (48.0 ± 12.2, 12–74)	12.6%	47	96	0
Multifocal dystonia	24 (33.3 ± 16.1, 7–67)	20.8%	8	16	0
Generalized dystonia	26 (23.3 ± 17.8, 1–57)	15.4%	12	14	0
Classified dystonias	1480		398	1082	9 (*P* = 0.0095)^3^
Unclassified dystonias^4^	192	NA	NA	NA	3
Dystonia total	1672				12 (*P* = 0.0035)^3^
Neurologically normal controls	1574 (61.2 ± 14.4, 23–95)	NA	688	886	1

NA, not available or applicable.

1 Mean age at study enrollment ± standard error, range (years).

2 First- or second-degree relative with dystonia.

3 Fisher's exact test for case–control analysis.

4 Subjects with unclassified dystonia were obtained from Athena Diagnostics.

### HRM and genotyping

High-resolution melting (HRM) was performed with the LightCycler® 480 real-time polymerase chain reaction (PCR) system and High Resolution Master Mix (Roche; Indianapolis, IN) in accordance with manufacturer instructions and our laboratory protocol (Xiao, et al. 2010). Melting curves and difference plots were analyzed using Gene Scanning Software. For samples with shifted melting curves, PCR products were cleaned using ExoSAP-IT® (United States Biochemical, Santa Clara, CA) and sequenced in the forward and reverse directions on an Applied Biosystems (Grand Island, NY) 3130XL Genetic Analyzer (Table S2).

### Extraction of RNA from leukocytes and cell lines

Ambion's (Grand Island, NY) LeukoLOCK™ Total RNA Isolation System and TRI Reagent® were used to isolate RNA from peripheral blood leukocytes of subjects with dystonia and controls. Using relative quantitative real-time reverse transcription PCR (QRT-PCR), we examined the effects of c.71+9C>A on *THAP1* gene expression in dystonia subjects harboring c.71+9C>A (*n* = 6) in comparison to controls without the variant (*n* = 12). For microarray, whole-genome gene expression studies, RNA was extracted from lymphoblastoid cell lines derived from six dystonia patients with c.71+9C>A and nine normal controls without this variant. Sanger sequencing was used to exclude coding, splice site, 5' untranslated region, and other previously reported intronic sequence variants (e.g., c.71+126T>C) from the controls.

To establish lymphoblastoid cell lines, peripheral blood mononuclear cells were separated by centrifugation on a sodium diatrizoate polysucrose gradient and transformed with Epstein–Barr virus (Coriell, Camden, NJ). The lymphoblastoid cell lines were propagated in Roswell Park Memorial Institute-1640 medium (Sigma-Aldrich, St. Louis, MO) supplemented with 15% fetal bovine serum (Sigma-Aldrich) and 2 mmol/L l-glutamine (Invitrogen, Grand Island, NY) at 37°C in 5% CO_2_. Confluent cells (1 × 10^6^ cells/mL) were harvested for RNA using TRI Reagent® from Ambion. The quality of total RNA derived from leukocytes and lymphoblastoid cell lines was accessed with a NanoDrop® ND-1000 spectrophotometer (NanoDrop Technologies, Wilmington, DE) and Agilent 2100 Bioanalyzer using the Agilent RNA 6000 Nano Chip kit. Subsequent downstream analyses were limited to samples with RNA Integrity Numbers greater than 8.0.

### Relative quantitative reverse transcription PCR

Reverse transcription was performed with Ambion's RETROscript™ kit using 500 ng of total RNA as template. The reaction mixture was incubated at 44°C for 1 hr followed by 10 min at 92°C. QRT-PCR was performed using the Roche LightCycler® 480 with specific primers for *THAP1* and *TOR1A* (Table S2), along with Universal ProbeLibrary (Roche) probes for human *THAP1*, *TOR1A*, and the endogenous control (cyclophilin D). Four endogenous controls (encoded proteins: cyclophilin D, *β*-tubulin, TATA-binding protein, hypoxanthine-guanine phoshoribosyltransferase) were examined. Cyclophilin D showed the smallest sample-to-sample variance. Student's *t*-test was used to compare RNA expression between dystonia and control samples.

### Whole-genome gene expression analysis

For microarray analyses, we used the Illumina® HumanHT-12 v.4 expression microarray platform (Illumina, San Diego, CA) to assess the expression levels in each individual sample. These arrays investigate whole-genome expression, providing coverage for more than 47,000 transcripts and known splice variants across the human transcriptome. In total, 15 samples, six from patient lymphoblastoid cell lines and nine from normal controls were processed on two chips. Total RNA (200 ng) was processed with the Illumina® TotalPrep™ RNA Amplification Kit (Applied Biosystems) to produce biotinylated complementary RNAs (cRNAs). This procedure included the reverse transcription of RNA to synthesize the first strand complementary DNA (cDNA), second-strand cDNA synthesis, cDNA purification, in vitro transcription to synthesize cRNA, cRNA amplification, and purification. The concentration of the cRNA solution was determined by measuring the absorbance at 260 nm/280 nm using the NanoDrop 1000A spectrophotometer. The biotinylated cRNAs (750 ng/sample) were hybridized to the arrays for 20 h at 58°C. These direct hybridized microarrays were scanned with the BeadArray® reader (Illumina, San Diego, CA). The raw data were processed for errors and quality checks using Illumina's proprietary GenomeStudio® software. Gene expression data were quartile normalized and summarized further with GeneSpring GX® 12.1 software (Agilent® Technologies, Santa Clara, CA). An unpaired *t*-test was done to filter probes that were significant at *P* ≤ 0.05 with a mean fold change ≥1.5. Filtered probes were further investigated for biological significance using WebGestalt Gene Set Analysis Toolkit 2.0 (http://bioinfo.vanderbilt.edu/webgestalt/) (Zhang et al. 2005). The differentially expressed gene set was compared to the human genome using the hypergeometric test followed by correction for multiple testing using the Benjamini and Hochberg (1995) (BH) method at a significance level of 0.05 (False Discovery Rate < 0.05). Kyoto Encyclopedia of Genes and Genomes (KEGG) pathways were accessed from WebGestalt (Ashburner et al. 2000; Kanehisa and Goto 2000). Dysregulated cellular networks were examined with Ingenuity Pathway Analysis (IPA; Ingenuity, Redwood City, CA).

### Minigene assay and in silico analysis of splicing

To test the effect of c.71+9C>A variant on splicing of *THAP1*, we performed a minigene assay. The wild-type minigene construct was produced by cloning into the exon trap vector pET01 (MoBiTec, Göttingen, Germany) in two steps. First, we introduced genomic DNA encompassing ∼400 bp 5′ to Exon 1, Exon 1, and ∼400 bp of Intron 1 into the pET01 vector. Genomic DNA was amplified with the SequalPrep™ Long PCR Kit from Invitrogen, followed by digestion of the PCR product and pET01 with XhoI and BamHI at 37°C for 2 h and then at 65°C for 20 min. This was followed by ligation of cohesive-ended gel-purified digested PCR products into pET01 with the LigaFast™ Rapid DNA Ligation System from Promega (Madison, WI). After sequence confirmation, the second part of the gene, which included ∼400 bp of Intron 1, Exon 2, Intron 2, and ∼600 bp of Exon 3, was introduced into the minigene construct with BamHI and NotI digestion (Table S3). After sequence confirmation, the variant c.71+9C>A was introduced into the wild-type minigene with the QuikChange® II XL Site-Directed Mutagenesis Kit from Stratagene (La Jolla, CA) to form the mutant minigene.

Human embryonic kidney (HEK293) cells were transfected in triplicates and total RNA was extracted after 72 h with TRI Reagent®. DNA was removed with DNase I (Ambion) and total RNA quality was examined with both agarose gel electrophoresis and a NanoDrop® ND-1000 spectrophotometer. Reverse transcription was performed with Ambion's RETROscript™ kit using 500 ng of total RNA as template. PCR products were examined on a 1% agarose gel, followed by quantifying the bands with ImageJ (http://rsbweb.nih.gov/ig/). Effects of genotype were evaluated with a Student's *t*-test.

The c.71+9C>A Intron 1 variant was further evaluated for its effects on splicing using a broad array of in silico programs which examine splicing, enhancer sites, and silencer sites. The programs used included Relative Enhancer and Silencer Classification by Unanimous Enrichment exonic enhancer site (RESCUE-ESE) (Fairbrother et al. 2002), Putative Exonic Splicing Enhancers/Silencers (PESX) (Zhang and Chasin 2004), Human Splicing Finder (HSF) (Desmet et al. 2009), NetGene2 (Brunak et al. 1991), Splice Site Prediction by Neural Network (NNSplice) (Reese et al. 1997), MaxEntScan (MES) (Yeo and Burge 2004), ESEfinder (Cartegni et al. 2003), and Automated Splice Site and Exon Definition Analyses (ASSAED) (Rogan et al. 1998).

### Flow cytometry cell-cycle analysis

For flow cytometry, 10 × 10^6^ lymphoblastoid cells were washed with phosphate-buffered saline (PBS) and fixed overnight with 90% ice-chilled ethanol at −20°C. Fixed cells were then washed with 3× PBS followed by incubation with propidium iodide staining buffer (PBS with RNaseA 100 *μ*g/mL and propidium iodide 50 *μ*g/mL) at 37°C for 30 min, and then overnight at 4°C in the dark. The analysis was performed with a BD Biosciences LSR II Flow Cytometer (San Jose, CA).

## Results

### Case–control analysis of *THAP1* c.71+9C>A frequency

The *THAP1* c.71+9C>A variant (rs200209986) was found in neurologically normal Caucasian controls with a MAF of 0.0318% (1/3148). This is comparable to reports from the 1000 Genomes project (MAF = 0.0916%, 2/2184) and EVS database (MAF = 0.199%, 26/13006). In subjects with dystonia, the MAF was significantly higher (*P* < 0.05) at 0.359% (12/3344) when compared with the 1000 Genomes project and neurologically normal Caucasian controls genotyped in this study. However, the difference in MAF did not reach statistical significance when compared with the EVS database (*P* = 0.13).

### Effects of c.71+9C>A on *THAP1* and *TOR1A* expression

In comparison to controls with no identified *THAP1* sequence variants, leukocytes that harbored the c.71+9C>A variant were associated with an elevated ratio of Isoform 1 (NM_018105, 3-exon variant) to Isoform 2 (NM_199003, 2-exon variant) (Table 2). However, the c.71+9C>A variant had no effect on overall expression of *THAP1*. In addition, the c.71+9C>A variant had no effect on expression of *TOR1A* in either lymphoblastoid cell lines or leukocytes (Table S4).

**Table 2:** *THAP1* mRNA expression in wild-type and mutant cells

	Isoform 1		Isoform 2		Isoform 1/Isoform 2	
			
Genotype/phenotype	Mean ± SEM	*P*^1^	Mean ± SEM	*P*^1^	Mean ± SEM	*P*^1^
Leukocytes						
Normal controls (*n* = 12)	14.99 ± 4.68	0.96	1.00 ± 0.33	0.29	14.99 ± 3.03	0.028
c.71+9C>A dystonia (*n* = 6)	14.93 ± 5.10		0.70 ± 0.27		21.25 ± 3.90	
Lymphoblastoid cell lines						
Normal controls (*n* = 12)	10.14 ± 0.41	0.19	1.06 ± 0.05	0.24	9.63 ± 0.30	0.99
c.71+9C>A dystonia (*n* = 6)	8.82 ± 0.83		0.93 ± 0.10		9.62 ± 0.34	

1 *P*-value of *t*-test statistic.

### *THAP1* c.71+9C>A is associated with transcriptional dysregulation

**Figure 1:**
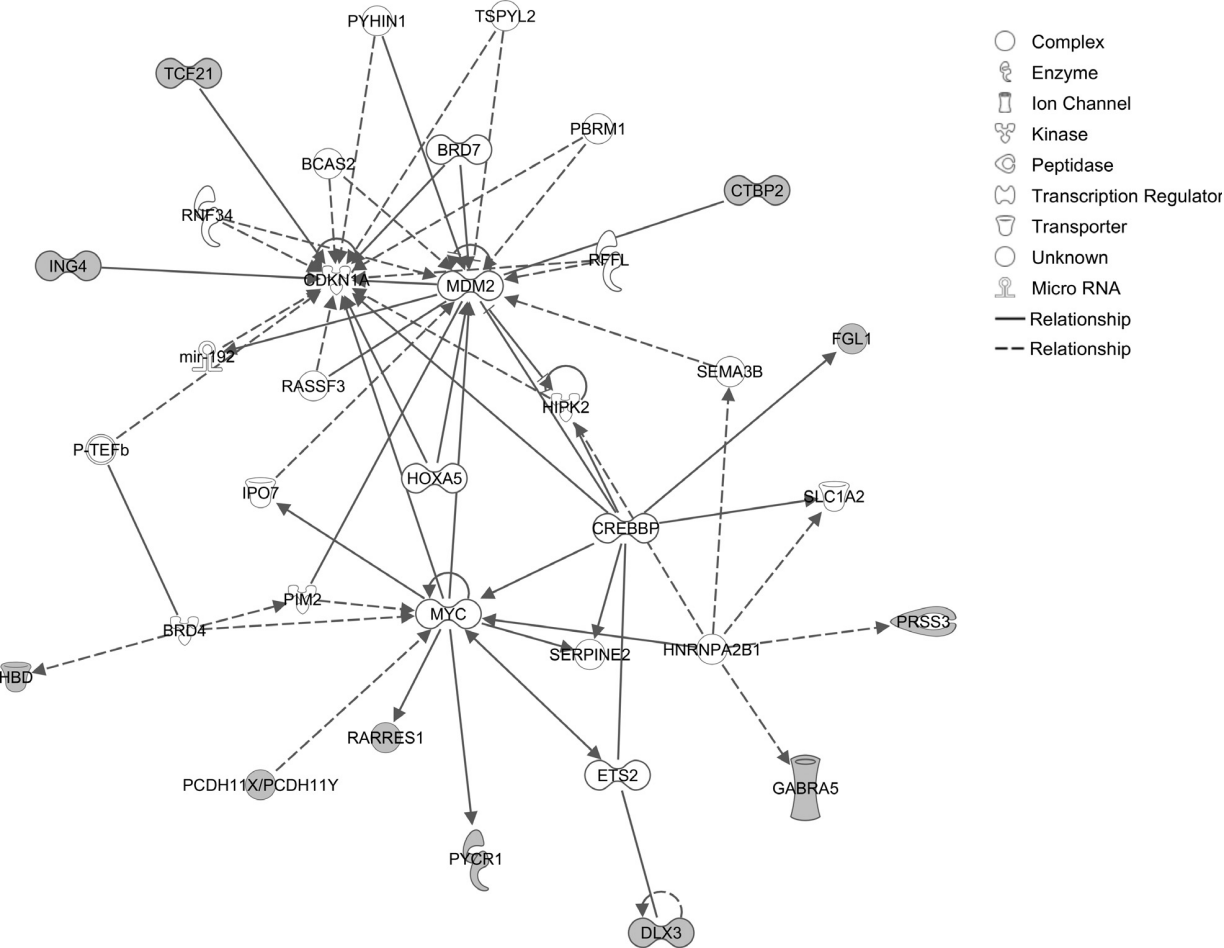
Ingenuity interactome analysis of upregulated genes.

**Figure 2:**
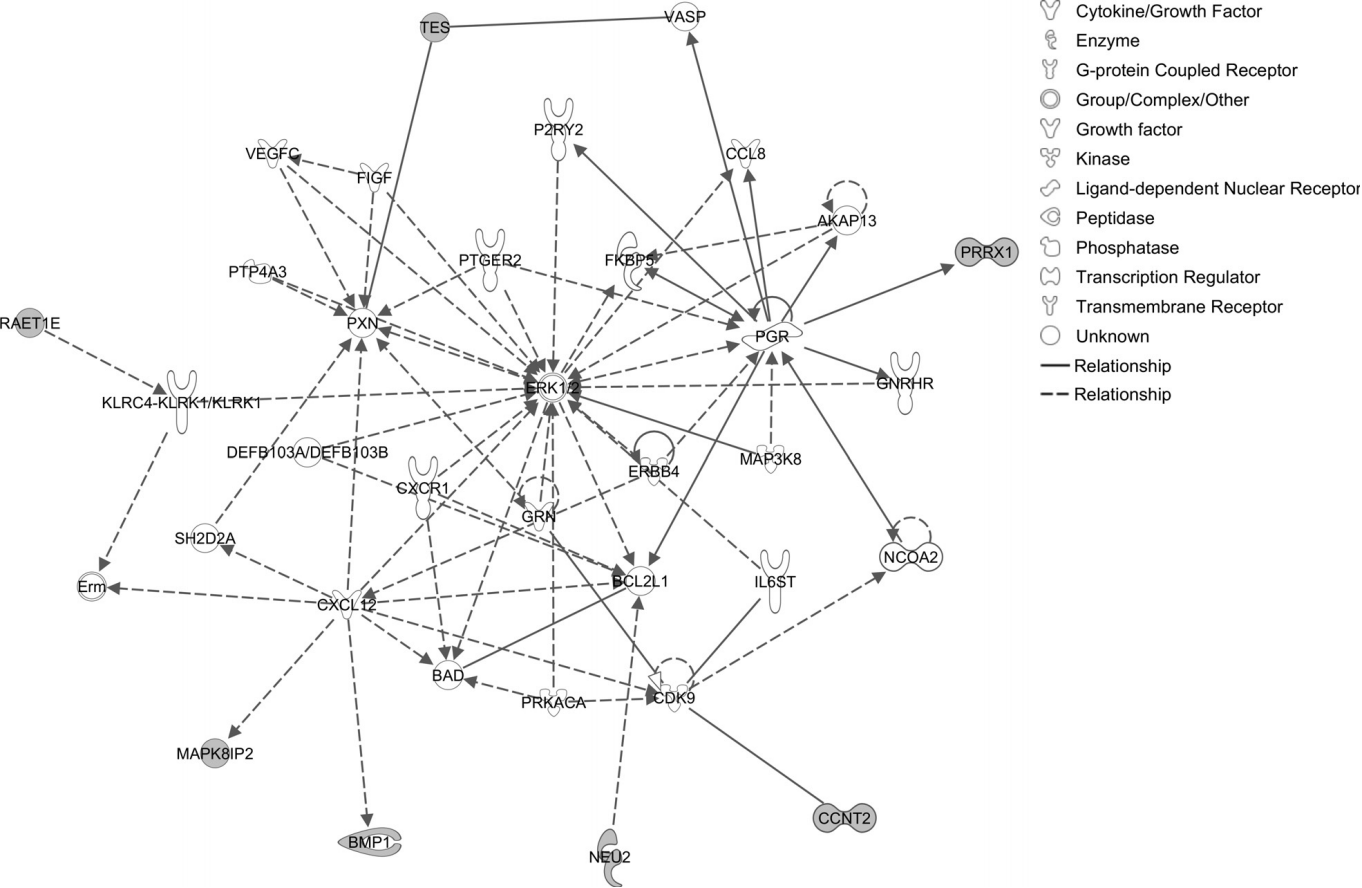
Ingenuity interactome analysis of downregulated genes.

Microarray gene expression analysis revealed significant upregulation of 234 genes; 277 genes were downregulated (Tables S5, S6). Gene set enrichment analysis using WebGestalt identified 29 significant KEGG pathways (Table S7). Upregulated pathways included cytokine–cytokine receptor interaction (*LIF*, *IL4R*, *CCL5*, *TGFBR2*, *LIFR*, *CCR2*, and *CXCL6*), chemokine signaling (*VAV3*, *CCL5*, *PLCB2*, *CCR2*, and *CXCL6*), natural killer cell-mediated cytotoxicity (*PPP3CA*, *VAV3*, *SH2D1A*, and *RAET1E*), WNT signaling (*CTBP2*, *PPP3CA*, *PLCB2*, and *WNT7A*), phophatidylinositol signaling (*PI4KA*, *DGKD*, and *PLCB2*), and JAK-STAT signaling (*LIF*, *LIFR*, *IL4R*, and *SOCS5*). Downregulated pathways included MAPK signaling (*ELK4*, *FGFR3*, *RAC1*, *CACNG6*, *MAPK8IP2*, and *PLA2G6*), endocytosis (*FGFR3*, *FOLR4*, *RNF41*, and *ADRBK2*), high-affinity receptor for the Fc region of immunoglobulin E and vascular endothelial growth factor (VEGF) signaling (*RAC1*, *PLA2G2F*, and *PLA2G6*) (Table S8). IPA interactome analysis of upregulated genes identified *CDKN1A*, *MDM2,* and *MYC* as hubs (Fig. 1). *ERK1/2* was a major hub for downregulated genes (Fig. 2).

Top canonical pathways upregulated, as indicated by IPA, included PI3K signaling. Top downregulated networks were involved in l-3, 4-dihydroxyphenylalanine degradation, glutamine degradation, and 1,25-dihydroxyvitamin D3 biosynthesis (Table S9).

### c.71+9C>A alters *THAP1* splicing

**Figure 3:**
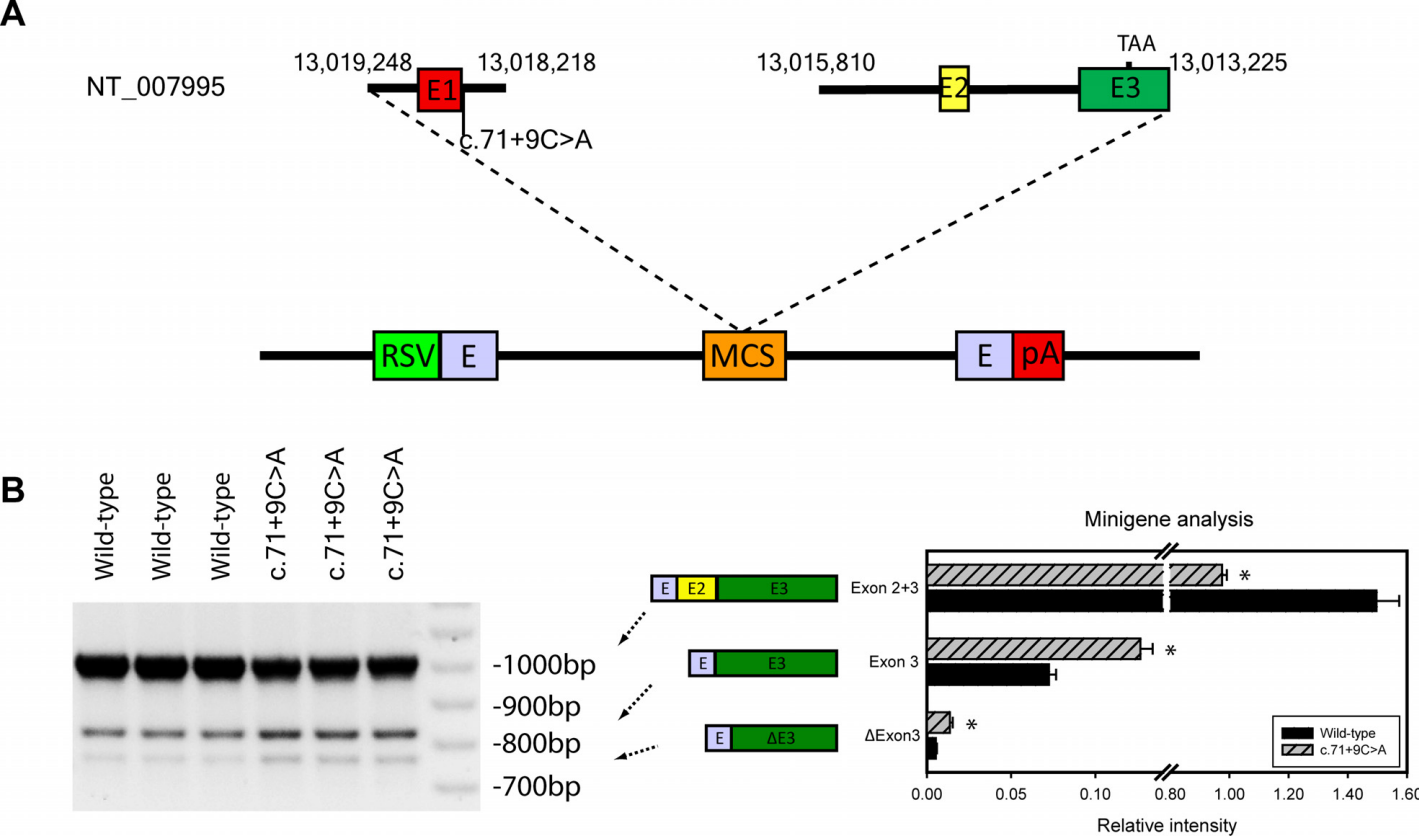
Minigene assay of *THAP1* splicing. (A) Segments of *THAP1* were cloned into the multiple cloning site (MCS) of the pET01 exon trap vector, which contains a short stretch of a eukaryotic phosphatase gene (E) 3′ to the eukaryotic strong long terminal repeater promoter of the Rous Sarcoma Virus (RSV). A second segment of E is followed by a polyadenylation site (pA). (B) The results of three wild-type and three c.71+9C>A transfections showed that the C>A mutation exerted significant effects on splicing patterns in HEK293 cells (*P < 0.05, for all).

Minigene results from three wild-type and three c.71+9C>A transfections showed that the c.71+9C>A variant exerted significant effects on splicing patterns (*P* < 0.05) (Fig. 3). Overall, mutant cells had reduced incorporation of Exon 2 and increased incorporation of Exon 3.

In silico analysis suggested that the c.71+9C>A variant may exert deleterious effects on transcription of *THAP1*. In aggregate, in silico analysis did not predict overt effects on splicing although the c.71+9C>A variant did alter enhancer locations. More specifically, HSF, NNSPLICE, MES, NetGene2 predicted no changes in splicing due to the mutant allele. On the other hand, ESEfinder, RESCUE-ESE, PESX, and ASSAED indicated that ESEs are altered by c.71+9C>A. RESCUE-ESE identified a new site, whereas PESX showed the wild-type ESE site as broken. ESEfinder indicated a drop in score from 93.79 to 90.90 for serine/arginine rich (SR) protein SC35. Other SR proteins, splicing factor 2/alternative splicing factor (SF2/ASF) and SRp40, were predicted to bind to new sites. ASSAED incurred changes in SC35 (6.7 bits to 6.0 bits), SRp40 (−1.0 bits to 5.3 bits), SF2/ASF (from 0.4 bits to 2.1 bits). All programs reported that exonic silencer sites (ESSs) remained unchanged by the c.71+9C>A variant.

### *THAP1* c.71+9C>A exerts effects on cell-cycle control

**Figure 4:**
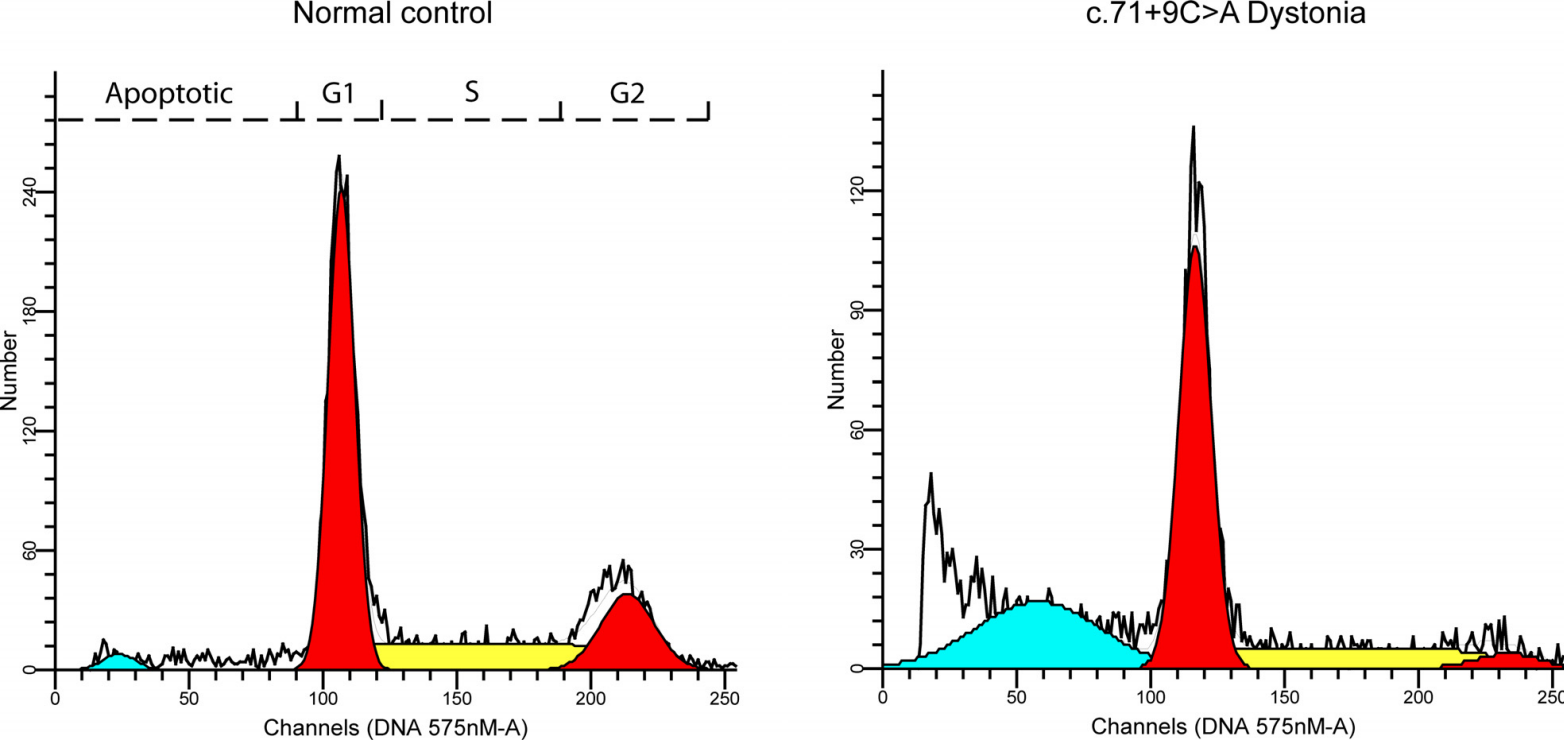
Cell-cycle analysis of lymphoblastoid cell lines. Data from representative normal and c.71+9C>A cells are shown. In comparison to normal cells, the proportion of c.71+9C>A cells in apoptotic phase is increased and G2 phase is decreased.

Cell-cycle analysis of control and c.71+9C>A lymphoblastoid cell lines showed overt differences (Fig. 4). Mutant variant cells, when compared with the normal wild-type genotypes, showed an increased percentage of apoptosis and decreased activity in G2 phase (Table S10).

## Discussion

Our study investigated the effects of a sequence variant (c.71+9C>A, rs200209986) located within Intron 1 of *THAP1* on gene splicing and gene expression in subjects with primary dystonia. The c.71+9C>A variant is rare in Caucasian populations with a MAF at less than 0.1% (1/3148) in our control cohort. This MAF is similar to data from the 1000 Genomes but lower than the MAF reported by the EVS database. In dystonia patients, the MAF (c.71+9C>A) was significantly higher than the controls of the 1000 Genomes project but not significantly higher than subjects in the EVS database. In other *THAP1* screening series, this variant was reported in 3/455 subjects with primary dystonia and 1/185 controls (Groen et al. 2010), 1/567 dystonia subjects and 1/365 controls (Lohmann et al. 2012), 0/109 dystonia subjects and 1/185 controls (Groen et al. 2011). In reports from other groups, it is not entirely clear if population controls had normal neurological examinations or a family history of any movement disorder including dystonia. Moreover, subjects recruited into EVS were not carefully examined to exclude dystonia, the diagnosis of which can be difficult even for an experienced general neurologist, often requiring the expertise of a movement disorders specialist. In addition, the EVS dataset is comprised of 2203 African-Americans and 4300 European-Americans, whereas our cohort was limited to Caucasians. The EVS cohort also included subjects with the extremes of specific traits and diseases including lipid disorders, hypertension, and coronary artery disease. Finally, sequence variants reported in the EVS dataset have not been validated with Sanger sequencing and some may be next-gen read errors (LeDoux 2012c). Therefore, case–control comparisons using the EVS cohort as a “control” group is problematic.

In vitro studies have identified a possible role for THAP1 in regulating the expression of *TOR1A* by binding to its promoter region (Gavarini et al. 2010; Kaiser et al. 2010). However, we did not detect an effect of variant *THAP1* on expression of *TOR1A* in either leukocytes or lymphoblastoid cell lines. Similarly, in previous work, RNAi knockdown of *THAP1* in fibroblasts showed no effect on expression levels of *TOR1A* (Gavarini et al. 2010). Overexpression of THAP1 in ECs has been used as an indirect means of identifying THAP1 targets – *TOR1A* was not among the 16 genes that were significantly upregulated or the 80 genes were downregulated (Cayrol et al. 2007). Consistent with these results, *THAP1* variants do not act as disease modifiers in DYT1 dystonia (Kamm et al. 2011).

The effects of c.71+9C>A on *THAP1* were studied with an integrated collection of in silico and in vitro approaches. Our in silico analysis showed that c.71+9C>A exerted significant effects on the exon splicing enhancer site, but not the splicing site. This finding is compatible with recent work showing that intronic variants can exert important effects on splicing enhancer sites (Fukao et al. 2010; Oeffner et al. 2011; Cohen et al. 2012; Jakubauskiene et al. 2012). Our in silico analysis was supported by a minigene assay which showed significant alterations in incorporation of Exons 2 and 3 in HEK293 cells transfected with the c.71+9C>A variant. In addition, leukocytes harboring the c.71+9C>A variant showed an elevated ratio of Isoform 1 (3-exon variant) to Isoform 2 (2-exon variant). This could represent a false positive, however, as we did not detect an effect of c.71+9C>A on splicing in lymphoblastoid cell lines. Although not entirely concordant, the minigene assay in HEK293 cells, in silico analyses, and QRT-PCR results in lymphoblastoid cell lines and leukocytes suggest that the c.71+9C>A variant may exert deleterious effects on splicing in human brain. In this regard, it is well-established that splicing shows tissue type and developmental specificity (Heinzen et al. 2008; Mondal et al. 2010).

Flow cytometry showed evidence of increased apoptosis and cell-cycle disruption in the variant lymphoblastoid cell lines. Previous studies defined a role for *THAP1* as a transcription factor binding to specific DNA sequences and regulating cell proliferation through pRB/E2F cell-cycle target genes (Clouaire et al. 2005; Cayrol et al. 2007). Furthermore, the pRB/E2F G1/S cell-cycle pathway appears to play a central role in the pathogenesis of dystonia, in general (LeDoux et al. 2013), and contributes to the apoptotic dopaminergic cell death associated with Parkinson's disease (Hoglinger et al. 2007).

The limitations of our functional analysis are well-recognized. Leukocytes and lymphoblastoid cell lines are not ideal surrogates for neural tissues. In this regard, the effects of genomic variants on splicing may be developmentally regulated and show tissue-type specificity. Ideally, the relative roles of the major and minor *THAP1* isoforms should be studied in a mammalian system via transgenic expression of the respective cDNAs on a *THAP1*-null background.

Gene expression data from lymphoblastoid cell lines provided evidence of significant transcriptional dysregulation associated with the c.71+9C>A variant. Upregulated pathways included cytokine–cytokine receptor interaction, chemokine signaling, cytotoxicity, Wnt signaling, and Jak-STAT signaling. Two genes that play a central role in the G1/S checkpoint pathway (*CDKN1A* and *MDM2*) were hubs in the interactome for upregulated genes. Conversely, downregulated pathways included MAPK signaling, endocytosis, and VEGF signaling. A critical element of MAPK signaling (extracellular signal-regulated kinase [ERK]1/2) was identified as a hub in the interactome for downregulated genes.

The differentially expressed individual genes identified in this study were mainly involved in cell division, communication, migration, and survival. The mitotic cell cycle is accomplished through a sequence of events that include DNA replication (S phase) and mitosis (M phase), separated by phases G1 and G2. This progression is regulated by cyclin-dependent kinases (CDKs). Any damage to the genome during these steps is corrected by repair pathways or arrest of progression from one stage to the next. Details from flow cytometry of c.71+9C>A lymphoblastoid cells indicate that apoptosis occurred prior to G1 with cells showing impairments in moving through the G1/S checkpoint and, as a result, a smaller percentage of cells were in the G2 phase. In a study by Cayrol et al. (2007), the overexpression of THAP1 was associated with inhibition of EC proliferation. Cell-cycle analysis of ECs transfected with *THAP1* siRNAs showed a reduction in both the S-phase and G2/M-phase cell populations and an increase in the number of cells in G1 thereby indicating the role of endogenous THAP1 in G1/S cell-cycle progression (Cayrol et al. 2007). Their data from both microarray and qPCR revealed that overexpression of THAP1 in primary HUVECs (human umbilical vein ECs) inhibits cell proliferation through coordinated repression of critical cell-cycle regulators and pRb/E2F target genes.

Upon phosphorylation, pRB is released, allowing the E2F transcription factor to interact with the transcriptional machinery of the cell, transcribing proteins crucial for G1/S progression and the S phase of the cell cycle. Phosphorylation of pRB is initiated by the cyclin D/Cdk4 and Cdk6 complex and continued by the cyclin E/Cdk2 complex. MAPK signaling, which was an enriched downregulated KEGG pathway in variant lymphoblastoid cells, activates Cyclin D/Cdk4 and Cdk6.

The MAPK signaling family plays an important role in complex cellular programs like proliferation, differentiation, development, transformation, and apoptosis. At least three MAPK pathways have been characterized: ERK, Jun kinase (JNK/SAPK) and p38 MAPK (Zhang and Liu 2002). ERK, when activated, will translocate to nucleus and change gene expression promoting differentiation or mitosis. ERK1/2 is involved in activating the microtubule-organizing center which controls the assembly of cytosolic microtubules in interphase cells and the mitotic spindle of dividing cells (Verlhac et al. 1993). ERK can translocate to the nucleus and phosphorylate transcription factors including ELK1, ELK4, and Tal. The ERK pathway can also link G0/G1 mitogenic signals to the immediate early response. The ERK pathway is known to be an intracellular checkpoint for cellular mitogenesis. The ERK cascade is a key in controlling the cell-cycle progression (Buchkovich and Ziff 1994). The Raf-MEK-ERK cascade regulates the posttranslational regulation of the assembly of cyclin D-Cdk4/6 complexes, which phosphorylate Rb protein causing activation of the E2F transcription factors that regulate the transcription of genes needed for G1/S transition (Blanchard et al. 2000).

Mean age of onset for THAP1-associated dystonia is 16.8 years and ranges from 3 to over 60 years (LeDoux et al. 2012). Although DYT1 dystonia is widely viewed as a neurodevelopmental circuit disorder (Xiao et al. 2004; Zhao et al. 2011), neurodegenerative processes may contribute to THAP1 and other mainly adult-onset dystonias (LeDoux et al. 2013). In recent work, the c.71+9C>A variant was associated with a significantly lower linear density of Purkinje cells in postmortem cerebellum from subjects with craniocervical dystonia and normal controls (Prudente et al. 2013). Other findings in subjects with craniocervical dystonia included ubiquitin-positive Marinesco bodies in the substantia nigra and areas of focal gliosis in cerebellar cortex. Transcriptional dysregulation in lymphoblastoid cells lines from subjects harboring the c.71+9C>A are compatible with these pathological findings and current themes in dystonia research which point to cerebellar Purkinje cells and nigrostriatal signaling as sites of functional abnormalities.

In the context of relative neurodevelopmental and neurodegenerative contributions to the pathogenesis of mainly adult-onset primary dystonia, Wnt, Jak-STAT, PI3K, and G1/S checkpoint pathways showed evidence of dysregulation in c.71+9C>A cells. Wnt pathways contribute to cell polarity, transcriptional regulation, and intracellular calcium homeostasis, all of which have been implicated in dystonia (LeDoux 2011; LeDoux et al. 2013). The JAK-STAT system is a cell-surface to nucleus signaling pathway leading to activation of genes that contribute to cell growth and differentiation. PI3K activates AKT which activates mammalian target of rapamycin (mTOR). As such the PI3K pathway is often referred to as the PI3K/AKT/mTOR pathway. The PI3K/AKT/mTOR pathway is central to the biology of glial tumors but also contributes to neural development (Wong 2013).

Given the apparent histological changes associated with c.71+9C>A postmortem human tissue and the results of IPA interactome analyses, the G1/S checkpoint pathway is a potential focal point of cellular pathology (Fig. 1). CDKN1A directly interacts with the dystonia-associated protein CIZ1 (Copeland et al. 2010). Moreover, other dystonia-associated proteins including THAP1, TAF1, ATM, and G*α*(olf), contribute to the G1/S checkpoint pathway (LeDoux et al. 2013). MDM2 is an E3 ubiquitin-protein ligase and negative regulator of p53. In theory, upregulation of CDKN1A and MDM2 could dampen the effects of THAP1 loss-of-function variants on G1/S cell-cycle control. Disturbances of the G1/S checkpoint could manifest as ultrastructural neurodevelopmental defects, apoptosis or both in terminally differentiated neurons such as Purkinje cells (Florio et al. 2012; Jurk et al. 2012).

## Supporting Information

**Table S1.** Phenotypes associated with the c.71+9C>A sequence variant. **Table S2.** Primers used for HRM, Sanger sequencing, and quantitative RT-PCR. **Table S3.** PCR primers used for the minigene assay. **Table S4.** *TOR1A* mRNA expression in leukocyte and lymphoblastoid cell lines. **Table S5.** Genes significantly upregulated in mutant cell lymphoblastoid lines. **Table S6.** Genes significantly downregulated in mutant cell lymphoblastoid lines. **Table S7.** Enriched KEGG pathways. **Table S8.** IPA top upregulated biological functions and canonical pathways. **Table S9.** IPA top downregulated biological functions and canonical pathways. **Table S10.** Cell-cycle analysis with propidium iodide.
