# Supplementary material for: A rare sequence variant in intron 1 of THAP1 is associated with primary dystonia
Source: Mol Genet Genomic Med. 2014 Feb 11;2(3):261–72. doi: 10.1002/mgg3.67 (PMC4049367; doi:10.1002/mgg3.67)
Supplement: Supplementary file 1 [file mgg30002-0261-SD1.docx]

**SUPPLEMENTARY FIGURES AND TABLES**

**A Rare Sequence Variant in Intron 1 of *THAP1* is Associated with Primary Dystonia**

Satya R. Vemula^1^, Jianfeng Xiao^1^, Yu Zhao^1^, Robert W. Bastian^2^, Joel S. Perlmutter^3^, Brad A. Racette^3^, Randal C. Paniello^4^, Zbigniew K. Wszolek^5^, Ryan J. Uitti^5^, Jay A. Van Gerpen^5^, Peter Hedera^6^, Daniel D. Truong^7^, Andrew Blitzer^8^, Monika Rudzińska^9^, Dragana Momčilović^10^, H. A. Jinnah^11^, Karen Frei^12^, Ronald F. Pfeiffer^1^, Mark S. LeDoux^1,*^

^1^Departments of Neurology and Anatomy & Neurobiology, University of Tennessee Health Science Center, Memphis, TN 38163, USA; ^2^Bastian Voice Institute, Downers Grove, IL, USA; ^3^Department of Neurology, Washington Univ. School of Medicine, St. Louis, MO, USA; ^4^Department of Otolaryngology-Head and Neck Surgery, Washington Univ. School of Medicine, St. Louis, MO, USA; ^5^Department of Neurology, Mayo Clinic, Jacksonville, FL, USA, 32224; ^6^Department of Neurology, Vanderbilt Univ., Nashville, TN, USA; ^7^Parkinson's & Movement Disorder Institute, Fountain Valley, CA, USA, 92708; ^8^New York Center for Voice and Swallowing Disorders, New York, NY, USA; ^9^Department of Neurology, Jagiellonian University Medical College in Krakow, Kraków, Poland; ^10^Clinic for Child Neurology and Psychiatry, Medical Faculty University of Belgrade, Belgrade, Serbia; ^11^Departments of Neurology, Human Genetics, and Pediatrics, School of Medicine, Emory University, Atlanta, GA 30322, USA; ^12^Loma Linda University Health System, Department of Neurology, Loma Linda, CA 92354

**Corresponding author:**

Mark S. LeDoux

University of Tennessee Health Science Center

Department of Neurology

855 Monroe Avenue, Link Building-Suite 415

Memphis, Tennessee 38163

Phone: (901) 448-6012

Fax: (901) 448-7440

E-mail: [mledoux@uthsc.edu](mailto:mledoux@uthsc.edu)

**Supp. Table S1. Phenotypes associated with the c.71+9C>A sequence variant**

| Subject  ID | Gender/Race | Age | Age of Onset | Family History | Site of Onset | Anatomical Classification |
| --- | --- | --- | --- | --- | --- | --- |
| P1 | F/C^a^ | 60 | 50 | Negative | Upper face | Focal (BSP) |
| P2 | M/C | 65 | 61 | Negative | Jaw | Focal (OMD) |
| P3 | F/C | 64 | 25 | Negative | Cervical | Focal (CD) |
| P4 | F/C | 66 | 58 | Negative | Cervical | Focal (CD) |
| P5 | M/C | 56 | 55 | Negative | Cervical | Focal (CD) |
| P6 | F/C | 62 | 48 | Negative | Larynx | Focal (SD) |
| P7 | F/C | 75 | 66 | Positive | Larynx | Focal (SD) |
| P8 | F/C | 60 | 58 | Positive | Larynx | Focal (SD) |
| P9 | M/C | 77 | 69 | Negative | Upper face | Focal (BSP) |
| P10 | NA | NA | NA | NA | NA | Unclassified |
| P11 | NA | NA | NA | NA | NA | Unclassified |
| P12 | NA | NA | NA | NA | NA | Unclassified |
| C1 | F/C | 55 | NA | NA | NA | Control |
| C2 | F/C | 50 | NA | NA | NA | Control |

^a^C, Caucasian of European origin. BSP, blepharospasm. OMD, oromandibular. CD, cervical dystonia.

SD, spasmodic dysphonia.

**Supp. Table S2. Primers used for HRM, Sanger sequencing and quantitative RT-PCR.**

| Primer name | Sequence (5’→3’) | Locus | Usage | Product (bp) |
| --- | --- | --- | --- | --- |
| THAP1_1F | aagaagcgagggaatccaac | NT_007995 13018721-702 |  |  |
| THAP1_1R | ccccaccccggctgaga | NT_007995 13018509-525 | HRM/Sanger | 213 (withTHAP1_1F) |
| THAP1_qr29F^a^ | agcttcccctgccttctg | NM_018105 185-202 |  |  |
| THAP1_qr29R^a^ | gactgggtcgagtaagaggaaa | NM_018105 331-310 | QRT-PCR (isoform 1) | 147 (with THAP1_qr29F) |
| THAP1_qr10F^a^ | cccgtttctttccacaaaaag | NM_199003 292-312 |  |  |
| THAP1_qr10R^a^ | agcatcaacctgggaaacag | NM_199003 383-364 | QRT-PCR (isoform 2) | 92 (with THAP1_qr10F) |
| TOR1A_qr25F^a^ | tcctaccagaaagccatgttc | NM_000113 666-686 |  |  |
| TOR1A_qr25F^a^ | gcttgatgtcttccctctgc | NM_000113 774-755 | QRT-PCR | 109 (with TOR1A_qr25F) |

^a^The number within the primer name corresponds to the probe number within the Roche Universal ProbeLibrary

**Supp. Table S3. PCR primers used for the minigene assay**

| Primer | Sequence (5’→3’)^a^ | Locus | Usage | Product (bp) |
| --- | --- | --- | --- | --- |
| THAP1_mut71+9F | ggccccgcgaggcgcgcag**T**gtcctcacttgtggaaagaaacgg | NT_007995 13018528-8571 | Mutagenesis |  |
| THAP1_mut71+9R | ccgtttctttccacaagtgaggac**A**ctgcgcgcctcgcggggcc | NT_007995 13018571-8528 | Mutagenesis |  |
| THAP1_miniF1 | **atatatctcgag**cactctgggaaaaggttcca | NT_007995 13019267-9248 |  |  |
| THAP1_miniR1 | **atatatggatcc**aaaacacctggctgctctgt | NT_007995 13018218-8237 | Minigene PCR1 | 1050 (with THAP1_miniF1) |
| THAP1_miniF2 | **atatatggatcc**cctttttcccagtacgcaga | NT_007995 13015829-5810 |  |  |
| THAP1_miniR2 | **atatatgcggccgc**ccaaaatccccaatcttgaa | NT_007995 13013225-3244 | Minigene PCR2 | 2605 (with THAP1_miniF2) |
| Exontrap_1F^b^ | cgccaagaacctcatcatct | Exontrap Vector 560-579 |  |  |
| Exontrap_1R^b^ | cgggacatgggtgtgtaga | Exontrap Vector 779-761 | Transfection  control | 220 (with Exontrap_1F) |
| Exontrap_2F | gagggatccgcttcctgcccc | Exontrap Vector 627-643 |  |  |
| THAP1_ENR | tgctggtacttcaactatttca | NT_007995 13013497-3518 | Minigene RT-PCR | Variable (with Exontrap_2F) |

^a^Nucleotides for site-directed mutagenesis are capitalized, and extra nucleotides for inclusion of restriction sites (underlined) are emboldened. ^b^Exontrap_1F and Exontrap_1R are localized to the long terminal repeat promoter of the Rouse Sarcoma Virus (RSV) and a eukaryotic gene (phosphatase) within the 5’ region of all transcripts generated by the minigene vector. Restriction enzyme sites are underlined.

**Supp. Table S4. *TOR1A* mRNA expression in leukocyte and lymphoblastoid cell lines**

| Genotype/Phenotype | Lymphoblastoid  cell lines | | Leukocytes | | |
| --- | --- | --- | --- | --- | --- |
|  | Mean ± SEM | *p*^a^ | Mean ± SEM | *p*^a^ | |
| Normal controls | 1.07 ± 0.13 (n = 12) | 0.91 | 1.01 ± 0.03 (n = 24) | | 0.30 |
| c.71+9C>A dystonia | 1.05 ± 0.13 (n = 6) |  | 1.08 ± 0.06 (n = 6) | |  |

^a^ *p*-value of *t*-test statistic

**Supp. Table S5. Genes significantly up-regulated in mutant cell lymphoblastoid lines**

| Gene Symbol | Gene Name and Description | Fold Change (FC≥2.0; *p≤*0.05) |
| --- | --- | --- |
| ATP2B4 | Atpase, Ca++ transporting, plasma membrane 4, transcript variant 2. | 16.27 |
| MMP9 | Matrix metallopeptidase 9 (Gelatinase B, 92 kDa Gelatinase, 92 kDa type IV Collagenase). | 12.33 |
| CRIM2 | Cysteine rich BMP regulator2 (Chordin-Like). | 9.56 |
| TGFBR2 | Transforming growth factor, beta receptor II (70/80 kDa), transcript variant 1. | 8.93 |
| FLJ44451 | Hypothetical protein flj44451 (Flj44451). | 8.09 |
| BANK1 | B-Cells scaffold protein with ankyrin repeats 1, transcript variant 2. | 7.99 |
| SPATA19 | Spermatogenesis associated 19. | 7.65 |
| ROBO2 | Roundabout, axon guidance receptor, homolog 2 (Drosophila). | 7.54 |
| SLC2A9 | Solute carrier family 2 (facilitated glucose transporter), member 9, transcript variant 1. | 7.38 |
| SNORD108 | Small nucleolar RNA, C/D box 108. | 7.25 |
| HTN3 | Histatin 3. | 7.23 |
| BHMT | Betaine-homocysteine S-methyltransferase. | 6.71 |
| MAGEA6 | Melanoma antigen family A, 6, transcript variant 2. | 6.70 |
| AGT | Angiotensinogen (serpin peptidase inhibitor, clade A, member 8). | 6.29 |
| PPARGC1A | Peroxisome proliferator-activated receptor gamma, coactivator 1 alpha. | 6.13 |
| SGEF | Src homology 3 domain containing guanine nucleotide exchange factor. | 6.13 |
| DKFZP564C196 | Dkfzp564C196 protein. | 6.08 |
| FLJ43950 | Flj43950 protein, transcript variant 1(Flj43950). | 6.02 |
| CEP192 | Centrosomal protein 192 kDa, transcript variant 2. | 6.00 |
| LAIR2 | Leukocyte-associated immunoglobulin like receptor 2, transcript variant 1. | 5.99 |
| FLJ46230 | Flj46230 protein (Flj46230). | 5.97 |
| FAM39DP | Family with sequence similarity 39, member D pseudogene on chromosome 15. | 5.91 |
| DGKD | Diacyl glycerol kinase, delta 130 kDa, transcript variant 1. | 5.90 |
| TAC3 | Tachykinin 3. | 5.86 |
| LTC4S | Leukotriene C4 synthase, transcript variant 2. | 5.77 |
| MIR1288 | MicroRNA 1288 (Mir1288), microRNA. | 5.69 |
| PSD | Pleckstrin and Sec 7 domain containing. | 5.67 |

***….Continued***

| Gene Symbol | Gene Name and Description | Fold Change (FC≥2.0; *p≤*0.05) |
| --- | --- | --- |
| SH3BGRL | SH3 domain binding glutamic acid rich protein like. | 5.64 |
| ZNF683 | Zinc finger protein 683, transcript variant 2. | 5.63 |
| ZNF33A | Zinc finger protein 33A, transcript variant 2. | 5.55 |
| SDS | Serine dehydratase. | 5.49 |
| SCIN | Scinderin. | 5.48 |
| CUBN | Cubilin (intrinsic factor cobalamin receptor). | 5.42 |
| SNORD116-27 | Small nucleolar RNA, C/D box 116-27, small nucleolar RNA. | 5.40 |
| MAFG | V-maf musculoaponeurotic fibrosarcoma oncogene homolog G, transcript variant 2. | 5.39 |
| ATAD5 | ATPase family, AAA domain containing 5. | 5.37 |
| TCF21 | Transcription factor 21, transcript variant 1. | 5.37 |
| OR12D3 | Olfactory receptor, family 12, subfamily D, member 3. | 5.34 |
| OPRS1 | Opioid receptor, sigma 1, transcript variant 5. | 5.33 |
| ACOT7 | Acyl-CoA thioesterase 7. | 5.31 |
| THRSP | Thyroid hormone responsive (spot 14 homolog, Rat). | 5.31 |
| MIR181B1 | MicroRNA 181 B-1, microRNA. | 5.30 |
| BRSK1 | BR serine/threonine kinase 1. | 5.29 |
| WDR78 | WD repeat domain 78, transcript variant 2. | 5.27 |
| KCND3 | Potassium voltage-gated channel, Shal-related subfamily, member 3, transcript variant 1. | 5.25 |
| DGCR10 | DiGeorge syndrome critical region gene 10, non-coding RNA. | 5.22 |
| SHF | Src homology 2 domain containing F. | 5.20 |
| LCE6A | Late cornified envelope 6A. | 5.17 |
| DIDO1 | Death inducer-obliterator 1, transcript variant 2. | 5.17 |
| KIAA0802 | Coiled-coil domain containing 165. | 5.16 |
| XAGE2 | X antigen family, member 2. | 5.11 |
| MGC48628 | Similar to kiaa1680 protein. | 5.07 |
| OPALIN | Oligodendrocytic myelin paranodal and inner loop protein, transcript variant 2. | 5.03 |
| SNCA | Synuclein, alpha (non A4 component of amyloid precursor). | 5.00 |
| ASZ1 | Ankyrin repeat, SAM and basic leucine zipper domain containing 1. | 4.98 |

***….Continued***

| Gene Symbol | Gene Name and Description | Fold Change (FC≥2.0; *p≤*0.05) |
| --- | --- | --- |
| TSPAN6 | Tetraspanin 6. | 4.97 |
| FAM90A17 | Family with sequence similarity 90, member A17. | 4.92 |
| CLPS | Colipase, pancreatic. | 4.92 |
| MRC1 | Mannose receptor, C type 1. | 4.83 |
| LPA | Lipoprotein, Lp (a). | 4.83 |
| LIFR | Leukemia inhibitory factor receptor alpha. | 4.82 |
| FAM179A | Family with sequence similarity 179, member A. | 4.80 |
| MIR920 | MicroRNA 920. | 4.74 |
| SNORD59A | Small nucleolar RNA, C/D box 59 A. | 4.74 |
| C18orf34 | Chromosome 18 open reading frame 34. | 4.74 |
| SLC16A4 | Solute carrier family16, member 4 (Mono carboxylic acid transporter 5). | 4.73 |
| KDM5A | Lysine(K)-specific demethylase 5A, transcript variant 2. | 4.72 |
| PCDH11X | Protocadherin 11 X-linked, transcript variant d. | 4.72 |
| CR1 | Complement component (3B/4B) receptor 1(Knops blood group) (Cr1). | 4.66 |
| MOCS1 | Molybdenum cofactor synthesis 1, transcript variant 4. | 4.66 |
| SPANXB1 | SPANX family, member B1. | 4.63 |
| DDO | D-Aspartate oxidase, transcript variant 2. | 4.62 |
| PRO0611 | Pro 0611 protein, non-coding RNA. | 4.58 |
| GPR77 | G protein-coupled receptor 77. | 4.54 |
| OR51G1 | Olfactory receptor, family 51, subfamily G, member 1. | 4.53 |
| SLC35F3 | Solute carrier family 35, member F3. | 4.53 |
| GBA2 | Glucosidase, Beta (bile acid) 2. | 4.52 |
| DNAH8 | Dynein, axonemal, heavy chain 8. | 4.52 |
| KNDC1 | Kinasenon-catalytic c-lobe domain (Kind) containing 1, transcript variant 1. | 4.49 |
| DBNDD2 | Dysbindin (dystrobrevin binding protein 1) domain containing 2, transcript variant 1. | 4.48 |
| FABP5L7 | Fatty acid binding protein 5-like 7. | 4.47 |
| RP1 | Retinitis pigmentosa 1(autosomal dominant). | 4.46 |
| LRRC66 | Leucine rich repeat containing 66. | 4.44 |

***….Continued***

| Gene Symbol | Gene Name and Description | Fold Change (FC≥2.0; *p≤*0.05) |
| --- | --- | --- |
| TELO2 | Tel2, telomere maintenance 2, homolog (*S.Cerevisiae*). | 4.44 |
| KCNJ6 | Potassium inwardly-rectifying channel, subfamily J, member 6. | 4.42 |
| HIST1H4D | Histone cluster 1, H4D. | 4.41 |
| C21orf126 | Chromosome 21 open reading frame 126. | 4.39 |
| ZNF107 | Zinc finger protein 107 (ZNF 107), transcript variant 2. | 4.37 |
| ATXN1L | Ataxin 1-like, transcript variant 2,Transcribedrna. | 4.37 |
| SLCO1A2 | Solute carrier organic anion transporter family, member 1 A2, transcript variant 1. | 4.34 |
| AFAR3 | Aflatoxin B1aldehyde reductase 3. | 4.32 |
| FER1L3 | Fer-1-like 3, myoferlin (*C.Elegans*), transcript variant 2. | 4.27 |
| CXCL6 | Chemokine (C-X-C motif) ligand 6 (Granulocyte chemotactic protein 2). | 4.27 |
| HBD | Hemoglobin, delta. | 4.26 |
| MIR1321 | MicroRNA 1321, micro RNA. | 4.23 |
| CILP | Cartilage intermediate layer protein, nucleotide pyrophospho hydrolase. | 4.18 |
| GABRA5 | Gamma-amino butyric acid (GABA) A receptor, alpha 5. | 4.16 |
| CTBP2 | C-terminal binding protein 2, transcript variant 1. | 4.15 |
| CRYBG3 | Beta-gamma crystalline domain containing 3. | 4.14 |
| C14orf162 | Chromosome 14 open reading frame 162. | 4.12 |
| CPA2 | Carboxy peptidase A2 (Pancreatic). | 4.09 |
| TM4SF19 | Transmembrane 4L six family member 19, transcript variant 3. | 4.08 |
| BRWD1 | Bromo domain and WD repeat domain containing 1, transcript variant 1. | 4.06 |
| SNX21 | Sorting nexin family member 21, transcript variant 4. | 4.02 |
| OSGIN1 | Oxidative stress induced growth inhibitor 1, transcript variant 1. | 4.02 |
| MIR548F1 | Micro RNA 548F-1, micro RNA. | 4.00 |
| FAM46D | Family with sequence similarity 46, memberd. | 3.99 |
| P2RY6 | Pyrimidinergic receptor P2Y, G-protein coupled, 6, transcript variant 3. | 3.98 |
| C11orf1 | Chromosome 11open reading frame 1. | 3.96 |
| MIR33B | MicroRNA 33B, microRNA. | 3.95 |

***….Continued***

| Gene Symbol | Gene Name and Description | Fold Change (FC≥2.0; *p≤*0.05) |
| --- | --- | --- |
| FAM13AOS | FAM 13A opposite strand (non-protein coding), non-coding RNA. | 3.94 |
| MIR141 | MicroRNA 141, micro RNA. | 3.94 |
| FREM2 | FRAS1 related extra cellular matrix protein 2. | 3.94 |
| PLXDC1 | PLEXIN domain containing 1. | 3.93 |
| CAPN10 | Calpain 10, transcript variant 8. | 3.92 |
| PPIAP19 | Peptidylprolyl isomerase A (cyclophilin A ) pseudogene 19. | 3.91 |
| N4BP1 | NEDD 4 binding protein 1. | 3.91 |
| MGAT5 | Mannosyl (alpha-1,6-)-glycoprotein beta-1,6-N-acetyl-glucosaminyltransferase. | 3.91 |
| MAP1B | Microtubule-associated protein 1B. | 3.90 |
| EFTUD1 | Elongation factor Tu GTP binding domain containing 1, transcript variant 1. | 3.88 |
| RGS12 | Regulator of G-protein signalling 12, transcript variant 5. | 3.88 |
| SLC13A3 | Solute carrier family 13 (Sodium-dependent dicarboxylate transporter), member 3, transcript variant 1. | 3.86 |
| HPS4 | Hermansky-Pudlak syndrome 4, transcript variant 3. | 3.84 |
| GK2 | Glycerol kinase 2. | 3.83 |
| DEFA5 | Defensin, alpha 5, Paneth cell-specific. | 3.82 |
| SFTPB | Surfactant protein B), transcript variant 2. | 3.82 |
| ARRDC1 | Arrestin domain containing 1. | 3.81 |
| C21orf130 | Chromosome 21 open reading frame 130, non-coding RNA. | 3.77 |
| NUPR1 | Nuclear protein, transcriptional regulator, 1, transcript variant 1. | 3.75 |
| CREB1 | Camp responsive element binding protein 1, transcript variant b. | 3.73 |
| ADAMTSL3 | ADAMTS-like3. | 3.73 |
| ARVP6125 | Misc_RNA. | 3.72 |
| HDX | Highly divergent homeo box. | 3.72 |
| TAS2R14 | Taste receptor, type 2, member 14. | 3.70 |
| ENTPD3 | Ectonucleoside triphosphate diphosphohydrolase 3. | 3.70 |
| C1orf68 | Chromosome 1, open reading frame 68. | 3.67 |
| SULT1E1 | Sulfotransferase family 1E, estrogen-preferring, member 1. | 3.65 |

***….Continued***

| Gene Symbol | Gene Name and Description | | Fold Change (FC≥2.0; *p≤*0.05) | |  |
| --- | --- | --- | --- | --- | --- |
| FCRL1 | | FC receptor-like 1. | | 3.61 | |
| FAM181B | | Family with sequence similarity 181, member B. | | 3.59 | |
| MYLK4 | | Myosin light chain kinase family, member 4. | | 3.58 | |
| C5orf46 | | Chromosome 5, open reading frame 46. | | 3.55 | |
| C3 | | Complement component 3. | | 3.55 | |
| SPTA1 | | Spectrin, alpha, erythrocytic 1 (Elliptocytosis 2). | | 3.53 | |
| FBXO9 | | F-Box protein 9, transcript variant 3. | | 3.53 | |
| CA12 | | Carbonic anhydrase XII, transcript variant 1. | | 3.52 | |
| PPP1R3B | | Proteinphosphatase 1, regulatory (Inhibitor) subunit 3B. | | 3.50 | |
| MIAT | | Myocardial infarction associated transcript (non-protein coding), non-coding RNA. | | 3.43 | |
| PRSS3 | | Protease, serine, 3 (mesotrypsin). | | 3.43 | |
| GATA6 | | GATA binding protein 6. | | 3.43 | |
| MYH4 | | Myosin, heavy chain 4, skeletal muscle. | | 3.42 | |
| CCR2 | | Chemokine (C-C motif) receptor 2, transcript variant b. | | 3.39 | |
| RSHL3 | | Radial spoke head-like 3. | | 3.38 | |
| LCE3E | | Late cornified envelope 3E. | | 3.38 | |
| IGFL1 | | IGF-like family member 1. | | 3.34 | |
| TMTC3 | | Transmembrane and tetratricopeptide repeat containing 3. | | 3.28 | |
| CAV3 | | Caveolin 3, transcript variant 1. | | 3.28 | |
| RBM3 | | RNA binding motif (Rnp1, Rrm) protein 3, transcript variant 2. | | 3.26 | |
| C10orf53 | | Chromosome 10, open reading frame 53, transcript variant 2. | | 3.24 | |
| PLCB2 | | Phospholipase C, beta 2. | | 3.16 | |
| PYCR1 | | Pyrroline-5-carboxylate reductase 1, transcript variant 1. | | 3.14 | |
| FLJ39632 | | Misc_RNA (Flj39632). | | 3.12 | |
| PDZD3 | | PDZ domain containing 3. | | 3.11 | |
| DLX3 | Distal-less homeo box 3. | | 3.10 | |  |
| WNT7A | Wingless-type MMTV integration site family, member 7A. | | 3.08 | |  |
| RARRES1 | Retinoic acid receptor responder (Tazarotene induced) 1, transcript variant 2. | | 3.08 | |  |

***….Continued***

| Gene Symbol | | Gene Name and Description | | Fold Change (FC≥2.0; *p≤*0.05) | |
| --- | --- | --- | --- | --- | --- |
| RAET1E | Retinoic acid early transcript 1E. | | 3.06 | |  |
| KRTAP4-12 | Keratin associated protein 4-12. | | 3.05 | |  |
| LY6G5C | Lymphocyte antigen 6 complex, locus G5C, transcript variant 1. | | 3.05 | |  |
| TRIM17 | Tripartite motif-containing 17, transcript variant 1. | | 3.03 | |  |
| CEACAM4 | Carcino embryonic antigen-related cell adhesion molecule 4. | | 3.00 | |  |
| MIR2115 | MicroRNA 2115. | | 2.99 | |  |
| SMAD1 | SMAD family member 1, transcript variant 1. | | 2.98 | |  |
| KCNK9 | Potassium channel, subfamily K, member 9. | | 2.98 | |  |
| ABHD11 | AB hydrolase domain containing 11, transcript variant 4. | | 2.96 | |  |
| FES | Feline sarcoma oncogene. | | 2.95 | |  |
| NTN5 | Netrin 5. | | 2.91 | |  |
| BCAR4 | Breast cancer anti-estrogen resistance 4, non-coding RNA. | | 2.89 | |  |
| C21orf41 | Chromosome 21, open reading frame 41. | | 2.89 | |  |
| MGC13005 | Hypothetical protein MGC 13005. | | 2.83 | |  |
| CHIC1 | Cysteine-rich hydrophobic domain 1. | | 2.73 | |  |
| C7orf20 | Chromosome 7, open reading frame 20, transcript variant 7. | | 2.68 | |  |
| ABHD1 | AB hydrolase domain containing 1. | | 2.60 | |  |
| SH2D1A | SH2 domain protein 1A, Duncan's disease (lympho proliferative syndrome). | | 2.59 | |  |
| STAR | Steroidogenic acute regulatory protein (STAR), nuclear gene encoding mitochondrial protein, transcript variant 2. | | 2.57 | |  |
| ING4 | Inhibitor of growth family, member 4 (ING 4), transcript variant 2. | | 2.51 | |  |
| RNF38 | Ring finger protein 38, transcript variant 5. | | 2.46 | |  |
| FGL1 | Fibrinogen-like 1, transcript variant 3. | | 2.45 | |  |
| CNGA4 | Cyclic nucleotide gated channel alpha 4. | | 2.36 | |  |
| ATP2B4 | ATPase, Ca++ transporting, plasmamembrane 4, transcript variant 1. | | 2.34 | |  |
| PPAP2B | Phosphatidic acid phosphatase type 2B, transcript variant 1. | | 2.34 | |  |
| KCNG2 | Potassium voltage-gated channel, subfamily G, member 2. | | 2.33 | |  |
| FANCA | Fanconi anemia, complementation group A, transcript variant 2. | | 2.31 | |  |

***….Continued***

| Gene Symbol | | Gene Name and Description | | Fold Change (FC≥2.0; *p≤*0.05) | |
| --- | --- | --- | --- | --- | --- |
| PRAMEF4 | PRAME family member 4. | | 2.28 | |  |
| ICAM5 | | Intercellular adhesion molecule 5, telencephalin. | | 2.17 | |
| ALX3 | | Aristaless-like homeobox 3. | | 2.14 | |
| PPIL5 | | Peptidylprolyl isomerase (cyclophilin)-like 5, transcript variant 2. | | 2.14 | |
| LIF | | Leukemia inhibitory factor (cholinergic differentiation factor). | | 2.10 | |
| F7 | | Coagulation factor VII (Serum prothrombin conversion accelerator), transcript variant 1. | | 2.07 | |
| SUZ12P | | Suppressor of zeste 12 homolog pseudogene, transcript variant 3(Suz12P). | | 2.04 | |
| VAV3 | | Vav 3 guanine nucleotide exchange factor, transcript variant 1. | | 2.00 | |

**Supp. Table S6. Genes significantly down-regulated in mutant cell lymphoblastoid lines**

| Gene Symbol | Gene Name and Description | Fold Change (FC≥2.0; *p≤*0.05) |
| --- | --- | --- |
| XRN2 | 5'-3' exoribonuclease 2. | 15.67 |
| ADAP2 | ArfGAP with dual PH domains 2. | 11.71 |
| C1orf115 | Chromosome 1, open reading frame 115. | 11.43 |
| TPRG1 | Tumor protein p63 regulated 1. | 11.18 |
| SIK1 | Salt-inducible kinase 1. | 8.64 |
| FBXO4 | F-box protein 4, transcript variant 2. | 8.37 |
| TFAP2A | Transcription factor AP-2 alpha (activating enhancer binding protein 2 alpha), transcript variant 3. | 8.21 |
| JSRP1 | Junctional sarcoplasmic reticulum protein 1). | 8.13 |
| M160 | Scavenger receptor cysteine-rich type 1 protein m 160. | 7.97 |
| SCARNA23 | Small Cajal body-specific RNA 23,Guide RNA. | 7.94 |
| PNMAL1 | PNMA-like 1, transcript variant 1. | 7.78 |
| EYA2 | Eyes absent homolog 2 (Drosophila), transcript variant 4. | 7.61 |
| CD33 | CD33 molecule, transcript variant 1. | 7.50 |
| KCNJ4 | Potassium inwardly-rectifying channel, subfamily J, member 4, transcript variant 1. | 7.46 |
| DZIP1 | Daz interacting protein 1, transcript variant 1. | 7.44 |
| GPR98 | G protein-coupled receptor 98, transcript variant 1. | 7.33 |
| DGCR6 | DiGeorge syndrome critical region gene 6. | 7.15 |
| MT1IP | Metallothionein1 I (pseudogene), non-coding RNA. | 6.78 |
| NMNAT3 | Nicotinamide nucleotide adenylyl transferase 3. | 6.74 |
| SEPT4 | Septin 4, transcript variant 1. | 6.64 |
| XGPY2 | XG pseudogene, Y-linked 2, non-coding RNA. | 6.55 |
| SLC25A41 | Solute carrier family 25, member 41. | 6.51 |
| FLJ30851 | Hypothetical loc 653140, transcript variant 3. | 6.21 |
| PCDH1 | Protocadherin 1, transcript variant 1. | 6.06 |
| OR4C3 | Olfactory receptor, family 4, subfamily C, member 3. | 6.04 |
| SNORA79 | Small nucleolar RNA, H/ACA box 79, small nucleolar RNA. | 6.01 |
| VCL | Vinculin, transcript variant 1. | 6.00 |

***….Continued***

| Gene Symbol | Gene Name and Description | | Fold Change (FC≥2.0; *p≤*0.05) | |  |
| --- | --- | --- | --- | --- | --- |
| FOLR4 | | Folate receptor 4 (Delta) homolog (Mouse). | | 5.91 | |
| ATP4A | | ATPase, H+/K+ exchanging, alpha polypeptide. | | 5.91 | |
| LRTOMT | | Leucine rich transmembrane and 0-methyltransferase domain containing. | | 5.91 | |
| PRSS8 | | Protease, serine, 8. | | 5.89 | |
| GPR126 | | G protein-coupled receptor 126, transcript variant a2. | | 5.88 | |
| CAPN6 | | Calpain 6. | | 5.73 | |
| MIR487A | | MicroRNA 487A. | | 5.67 | |
| DLL3 | | Delta-like 3 (Drosophila), transcript variant 1. | | 5.64 | |
| MTMR11 | Myotubularin related protein 11. | | 5.55 | |  |
| SPG7 | Spastic paraplegia 7 (Pure and complicated autosomal recessive), nuclear gene encoding mitochondrial protein, transcript variant 2. | | 5.52 | |  |
| C21orf34 | Chromosome 21, open reading frame 34, transcript variant 3. | | 5.46 | |  |
| CKMT1A | Creatine kinase, mitochondrial 1A, nuclear gene encoding mitochondrial protein. | | 5.44 | |  |
| GZF1 | GDNF-inducible zinc finger protein 1. | | 5.37 | |  |
| IGHMBP2 | Immunoglobulin mu binding protein 2. | | 5.36 | |  |
| HMGA1 | High mobility group at-hook 1, transcript variant 4. | | 5.36 | |  |
| MIR100 | Micro RNA 100. | | 5.33 | |  |
| ADAM12 | ADAM metallopeptidase domain 12 (Meltrin alpha), transcript variant 1. | | 5.28 | |  |
| RMRP | RNA component of mitochondrial RNA processing endoribonuclease. | | 5.23 | |  |
| MIR122 | Micro RNA 122. | | 5.19 | |  |
| C3orf54 | Chromosome 3, open reading frame 54. | | 5.16 | |  |
| LUZP6 | Leucine zipper protein 6. | | 5.13 | |  |
| TFAP2A | Transcription factor AP-2 alpha (Activating enhancer binding protein 2 alpha), transcript variant 1. | | 5.11 | |  |
| C17orf58 | Chromosome 17, open reading frame 58, transcript variant 2. | | 5.10 | |  |
| FBXO43 | F-Box protein 43, transcript variant 2. | | 5.08 | |  |
| TSP50 | Testes-specific protease 50. | | 5.05 | |  |
| TMEM120B | Transmembrane protein 1 20B. | | 4.96 | |  |
| C14orf178 | Chromosome 14, open reading frame 178. | | 4.94 | |  |

***….Continued***

| Gene Symbol | Gene Name and Description | Fold Change (FC≥2.0; *p≤*0.05) |
| --- | --- | --- |
| CHRNA10 | Cholinergic receptor, nicotinic, alpha10. | 4.92 |
| FNIP1 | Folliculin interacting protein 1, transcript variant 1. | 4.85 |
| C19orf71 | Chromosome 19, open reading frame 71. | 4.81 |
| BEGAIN | Brain-enriched guanylate kinase-associated homolog (Rat). | 4.81 |
| MIXL1 | MIX1 homeobox-like 1(*Xenopus laevis*). | 4.77 |
| FLJ46257 | Flj46257 protein. | 4.75 |
| CYP4B1 | Cytochrome p450, family 4, subfamily B, polypeptide 1. | 4.73 |
| SPANXE | SPANX family, member E. | 4.71 |
| RGS22 | Regulator of G-protein signalling 22. | 4.70 |
| MYRIP | Myosin VIIA and Rab interacting protein, transcript variant 3. | 4.70 |
| ZPBP2 | Zona pellucida binding protein 2, transcript variant 1. | 4.69 |
| ZNF16 | Zinc finger protein 16, transcript variant 1. | 4.68 |
| C1orf117 | Chromosome 1, open reading frame 117. | 4.67 |
| MESP2 | Mesoderm posterior 2 homolog (Mouse). | 4.66 |
| PLA2G2F | Phospholipase A2, group IIF. | 4.66 |
| FOXP2 | Fork head box P2, transcript variant 1. | 4.66 |
| RBL1 | Retinoblastoma-like 1(P107), transcript variant 2. | 4.64 |
| SMR3A | Submaxillary gland androgen regulated protein 3A. | 4.64 |
| ZNF197 | Zinc finger protein 197, transcript variant 2. | 4.63 |
| CYP2D7P1 | Cytochrome p450, family 2, subfamily D, polypeptide 7 pseudogene 1, miscRNA. | 4.63 |
| SNORD50B | Small nucleolar RNA, C/D box 50B, small nucleolar RNA. | 4.63 |
| C5orf45 | Chromosome 5, open reading frame 45, transcript variant 2. | 4.63 |
| TAAR8 | Trace amine associated receptor 8. | 4.60 |
| BAIAP3 | Bai1-associated protein 3. | 4.54 |
| FUNDC2 | Fun 14 domain containing 2. | 4.54 |
| MICALL2 | Mical-like 2, transcript variant 1. | 4.52 |
| EYA2 | Eyes absent homolog 2 (Drosophila), transcript variant 1. | 4.49 |
| L3MBTL3 | L (3) Mbt-like3 (Drosophila), transcript variant 2. | 4.49 |

***….Continued***

| Gene Symbol | Gene Name and Description | Fold Change (FC≥2.0; *p≤*0.05) |
| --- | --- | --- |
| SYCP1 | Synaptonemal complex protein 1. | 4.48 |
| FLJ41941 | Hypothetical loc 100192420, non-coding RNA. | 4.47 |
| CBWD1 | Cob WD domain containing 1, transcript variant 1. | 4.46 |
| FRYL | Fry-Like. | 4.45 |
| MIR204 | Micro RNA 204. | 4.45 |
| PSPH | Phosphoserine phosphatase. | 4.42 |
| SLIT3 | Slit homolog 3 (Drosophila3). | 4.39 |
| DHRS4L1 | Dehydrogenase/Reductase (Sdr family) member 4 like 1. | 4.38 |
| SNORA52 | Small nucleolar RNA, H/Aca box 52, small nucleolar RNA. | 4.37 |
| ACVRL1 | Activin A receptor type II-like 1, transcript variant 1. | 4.36 |
| ZNF192 | Zinc finger protein 192. | 4.33 |
| MIR544 | Micro RNA 544. | 4.33 |
| C21orf136 | Chromosome 21, open reading frame 136. | 4.32 |
| BMF | BCL2 modifying factor, transcript variant 2. | 4.32 |
| KRT20 | Keratin 20. | 4.32 |
| UBE2NL | Ubiquitin-conjugating enzyme e2N-like. | 4.30 |
| SEMG2 | Semenogelin II. | 4.30 |
| TMCO5A | Transmembrane and coiled-coil domains 5A. | 4.28 |
| MAPK8IP2 | Mitogen-activated protein kinase8 interacting protein 2, transcript variant 2. | 4.28 |
| THAP9 | THAP domain containing 9. | 4.26 |
| KLF3 | Kruppel-like factor 3 (Basic). | 4.25 |
| C6orf27 | Chromosome 6, open reading frame 27. | 4.23 |
| TES | Testis derived transcript (3 Lim domains), transcript variant 2. | 4.21 |
| RAC1 | Ras-related C3 botulinum toxin substrate 1 (Rho family, small GTP binding protein RAC1), transcript variant rac1B. | 4.20 |
| SDR42E1 | Short chain dehydrogenase/reductase family 42E, member 1. | 4.20 |
| THEM5 | Thioesterase super family member 5. | 4.19 |
| RAET1E | Retinoic acid early transcript 1E. | 4.18 |

***….Continued***

| Gene Symbol | Gene Name and Description | Fold Change (FC≥2.0; *p≤*0.05) |
| --- | --- | --- |
| ARMC8 | Armadillo repeat containing 8, transcript variant 2. | 4.17 |
| ADRBK2 | Adrenergic, beta, receptor kinase 2. | 4.17 |
| CSMD3 | Cub and sushi multiple domains 3, transcript variant c. | 4.17 |
| SLCO3A1 | Solute carrier organic anion transporter family, member 3A1. | 4.17 |
| CDH17 | Cadherin 17, LI cadherin (Liver-Intestine). | 4.13 |
| BMP1 | Bone morphogenetic protein 1, transcript variant bmp1-4. | 4.12 |
| SPO11 | Spo11 meiotic protein covalently bound to DSB homolog (*S.Cerevisiae*), transcript variant 1. | 4.06 |
| TMPO | Thymopoietin, transcript variant 2. | 4.04 |
| GC | Group-specific component (Vitamin D binding protein mRNA. | 4.04 |
| PRRX1 | Paired related homeobox 1, transcript variant pmx-1A. | 4.03 |
| ACVR1B | Activina receptor,Type Ib, transcript variant 3. | 4.02 |
| KRTAP4-11 | Keratin associated protein 4-1. | 4.02 |
| SLC16A1 | Solute carrier family 16, member 1 (Monocarboxylic acid transporter 1), transcript variant 1. | 4.02 |
| DHODH | Dihydroorotate dehydrogenase, nuclear gene encoding mitochondrial protein. | 3.99 |
| FLJ40672 | Hypothetical protein loc 732283. | 3.98 |
| SCRT1 | Scratch homolog 1, Zinc finger protein (Drosophila). | 3.96 |
| LFNG | LFNG O-fucosylpeptide 3-beta-N-acetylglucosaminyltransferase, transcript variant 2. | 3.96 |
| HRASLS | Hras-like suppressor. | 3.92 |
| C6orf176 | Misc_RNA. | 3.92 |
| NKAPL | Nfkb activating protein-like. | 3.91 |
| FAM25C | Family with sequence similarity 25, member C. | 3.91 |
| DNAHL1 | Dynein, axonemal, heavy chain like 1. | 3.89 |
| WBP11P1 | WW domain binding protein 11pseudogene 1, non-coding RNA. | 3.88 |
| TMCO2 | Transmembrane and coiled-coil domains 2. | 3.86 |
| C2orf40 | Chromosome 2, open reading frame 40. | 3.86 |
| NDST4 | N-Deacetylase/N-sulfotransferase (Heparanglucosaminyl) 4. | 3.85 |
| GLIS3 | GLIS family zinc finger 3, transcript variant 2. | 3.84 |

***….Continued***

| Gene Symbol | Gene Name and Description | Fold Change (FC≥2.0; *p≤*0.05) |
| --- | --- | --- |
| MIR367 | Micro RNA 367. | 3.83 |
| CCDC135 | Coiled-coil domain containing 135. | 3.82 |
| NCRNA00160 | Non-protein coding rna 160, non-coding RNA. | 3.82 |
| ZNF385B | Zinc finger protein 385B, transcript variant 1. | 3.81 |
| KIF27 | Kinesin family member 27. | 3.81 |
| HEPN1 | Hepacamopposite strand 1. | 3.80 |
| B3GNT4 | UDP-GlcNac:betaGal beta-1,3-n-acetylglucosaminyltransferase4. | 3.77 |
| MYO19 | Myosin XIX, transcript variant 3. | 3.74 |
| TTTY7 | Testis-specific transcript,Y-linked 7 (Non-protein coding), non-coding RNA. | 3.70 |
| APOA1 | Apolipoprotein A-I. | 3.69 |
| C10orf41 | Chromosome 10, open reading frame 41. | 3.67 |
| MIR643 | Micro RNA 643. | 3.65 |
| CSNK1A1P | Casein kinase 1, alpha 1 pseudogene, non-coding RNA. | 3.64 |
| PEMT | Phosphatidylethanolaminen-methyltransferase, nuclear gene encoding mitochondrial protein, transcript variant 2. | 3.64 |
| MIR132 | Micro RNA 132. | 3.63 |
| SPRR2D | Small proline-rich protein 2D. | 3.62 |
| NCRNA00051 | Non-protein coding RNA 51. | 3.61 |
| POP5 | Processing of precursor 5, ribonuclease P/ Mrp subunit (*S.Cerevisiae*), transcript variant 2. | 3.61 |
| MIR548I1 | Micro RNA 548I-1. | 3.61 |
| CACNB2 | Calcium channel, Voltage-dependent, beta 2 subunit, transcript variant 3. | 3.60 |
| TBC1D21 | Tbc1 domain family, member 21. | 3.59 |
| FGFR3 | Fibroblast growth factor receptor 3 (Achondroplasia, Thanatophoric dwarfism), transcript variant 1. | 3.57 |
| RSPH10B | Radial spoke head 10 homolog B (Chlamydomonas). | 3.52 |
| TNFRSF25 | Tumor necrosis factor receptor superfamily, member 25, transcript variant 12. | 3.52 |
| CDC2L1 | Cell division cycle2-like 1(pitsire proteins), transcript variant 3. | 3.52 |
| BAI1 | Brain-specific angiogenesis inhibitor 1. | 3.46 |
| CLRN2 | Clarin 2. | 3.45 |

***….Continued***

| Gene Symbol | Gene Name and Description | Fold Change (FC≥2.0; *p≤*0.05) |
| --- | --- | --- |
| PJA1 | Praja ring finger 1, transcript variant 1. | 3.43 |
| CACNG6 | Calcium channel, voltage-dependent, gamma subunit 6, transcript variant 3. | 3.42 |
| MUC3A | Mucin 3A, cell surface associated. | 3.42 |
| NEU2 | Sialidase 2 (Cytosolicsialidase). | 3.41 |
| FLJ35934 | Flj35934 Protein. | 3.35 |
| WFDC10B | WAP four-disulfide core domain 10B, transcript variant 1. | 3.35 |
| CD163 | Cd163 molecule, transcript variant 2. | 3.32 |
| SULT1A4 | Sulfotransferase family, cytosolic, 1A, phenol-preferring, member 4, transcript variant 2. | 3.31 |
| MID1 | Midline 1(Opitz/Bbb syndrome), transcript variant 3. | 3.30 |
| SNORD37 | Small nucleolar RNA, C/D box 37. | 3.30 |
| ZNF566 | Zinc finger protein 566. | 3.30 |
| FLJ41423 | Flj41423 protein. | 3.29 |
| SMARCD1 | Swi/Snf related, matrix associated, actin dependent regulator of chromatin, subfamily D, member 1, transcript variant 1. | 3.27 |
| C19orf28 | Chromosome 19, open reading frame 28, transcript variant 2. | 3.27 |
| NDUFC2 | Nadh dehydrogenase (Ubiquinone)1, sub complex unknown, 2, 14.5 kDa. | 3.26 |
| NOX5 | Nadph oxidase, EF-hand calcium binding domain 5. | 3.25 |
| ZCCHC5 | Zinc finger, CCHC domain containing 5. | 3.25 |
| NAT1 | N-acetyltransferase 1 (arylaminen-acetyltransferase). | 3.23 |
| RGPD3 | Ranbp2-like and grip domain containing 3, transcript variant 1. | 3.20 |
| C14orf180 | Chromosome 14, open reading frame 180. | 3.14 |
| PLCB3 | Phospholipasec, beta 3 (phosphatidylinositol-specific). | 3.12 |
| LHX4 | Lim homeobox 4. | 3.10 |
| OR1F1 | Olfactory receptor, family 1, subfamily F, member 1. | 3.10 |
| FLJ32214 | Hypothetical protein flj32214. | 3.07 |
| MIR126 | Micro RNA 126. | 3.07 |
| SLC38A11 | Solute carrier family 38, member 11. | 3.06 |
| ZNF197 | Zincfingerprotein197, transcript variant 1. | 3.02 |

***….Continued***

| Gene Symbol | Gene Name and Description | Fold Change (FC≥2.0; *p≤*0.05) |
| --- | --- | --- |
| C21orf131 | Chromosome21 open reading frame 131, non-coding RNA. | 3.02 |
| SLC44A4 | Solute carrier family 44, member 4. | 3.01 |
| ELK4 | Elk4, Ets-domain protein (Srf accessory protein 1), transcript variant a. | 2.98 |
| OSCAR | Osteoclast associated, immunoglobulin-like receptor, transcript variant 3. | 2.97 |
| CYLC1 | Cylicin, basic protein of sperm head cytoskeleton 1, transcript variant 2. | 2.95 |
| PCDHB14 | Protocadherin beta 14. | 2.95 |
| DDX12 | Dead/H (Asp-Glu-Ala-Asp/His) Box polypeptide 12 (Chl1-like helicase homolog, *S.Cerevisiae*), transcript variant 3. | 2.94 |
| FLJ46321 | Flj46321 protein. | 2.93 |
| KRTAP3-2 | Keratin associated protein 3-2. | 2.93 |
| C1orf95 | Chromosome 1, open reading frame 95. | 2.82 |
| HSD17B3 | Hydroxysteroid (17-beta) dehydrogenase 3. | 2.82 |
| KBTBD10 | Kelch repeat and BTB (POZ) domain containing 10. | 2.80 |
| C2orf48 | Chromosome 2, open reading frame 48. | 2.79 |
| FCN1 | Ficolin (collagen/fibrinogen domain containing) 1. | 2.77 |
| PFTK2 | PFTAIRE protein kinase 2. | 2.76 |
| ZNF70 | Zincfingerprotein70. | 2.68 |
| MIR1262 | Micro RNA 1262. | 2.67 |
| ZCCHC10 | Zinc finger, CCHC domain containing 10. | 2.67 |
| YIPF4 | Yip1 domain family, member 4. | 2.65 |
| ITM2A | Integral membrane protein 2A. | 2.65 |
| HECW1 | Hect, C2 and WW domain containing E3 Ubiquitin protein ligase 1. | 2.64 |
| C1orf58 | Chromosome 1, open reading frame 58. | 2.52 |
| FAM169A | Family with sequence similarity 169, member A. | 2.50 |
| CCNT2 | Cyclin T2, transcript variant b. | 2.47 |
| FLJ40296 | Flj40296 protein. | 2.46 |
| C20orf134 | Chromosome 20, open reading frame 134). | 2.45 |
| AGR3 | Anterior gradient homolog 3 (*Xenopus laevis*). | 2.44 |

***….Continued***

| Gene Symbol | Gene Name and Description | Fold Change (FC≥2.0; *p≤*0.05) |
| --- | --- | --- |
| SLC6A13 | Solute carrier family 6 (Neuro transmitter transporter, GABA),Member13. | 2.40 |
| MIR891A | Micro RNA 891A. | 2.37 |
| GAGE12J | G antigen 12J. | 2.35 |
| ZNF92 | Zinc finger protein 92, transcript variant 1. | 2.31 |
| SCAND2 | Scan domain containing 2 on chromosome15. | 2.24 |
| ZNF436 | Zinc finger protein 436, transcript variant 2. | 2.10 |
| DQX1 | DEAQ box polypeptide 1(RNA-dependentatpase). | 2.06 |
| PHF7 | Phd finger protein 7, transcript variant 2. | 2.06 |
| BRD4 | Bromo domain containing 4, transcript variant long. | 2.02 |
| C6orf160 | Chromosome 6, open reading frame 160, transcript variant 4. | 2.02 |
| GLS2 | Glutaminase 2 (liver, mitochondrial), nuclear gene encoding mitochondrial protein. | 2.01 |
| C2orf18 | Chromosome 2, open reading frame 18. | 2.01 |

**Supp. Table S7. Enriched KEGG pathways**

| Pathway | No. of Genes | Enrichment^a^ | raw *P*-value^b^ | adj *P*-value^c^ |
| --- | --- | --- | --- | --- |
| *Up-regulated* |  |  |  |  |
| Metabolic pathways | 12 | 2.23 | 8.2E-03 | 3.6E-02 |
| Cytokine-cytokine receptor interaction | 7 | 5.56 | 3.0E-04 | 1.4E-02 |
| Chemokine signaling pathway | 5 | 5.57 | 2.2E-03 | 2.1E-02 |
| Pancreatic secretion | 4 | 8.33 | 1.4E-03 | 2.1E-02 |
| Natural killer cell mediated cytotoxicity | 4 | 6.19 | 4.1E-03 | 3.0E-02 |
| Wnt signaling pathway | 4 | 5.61 | 5.9E-03 | 3.2E-02 |
| Jak-STAT signaling pathway | 4 | 5.43 | 6.6E-03 | 3.2E-02 |
| Glycerolipid metabolism | 3 | 12.62 | 1.8E-03 | 2.1E-02 |
| Complement and coagulation cascades | 3 | 9.15 | 4.4E-03 | 3.0E-02 |
| Phosphatidylinositol signaling system | 3 | 8.09 | 6.2E-03 | 3.2E-02 |
| Fc gamma R-mediated phagocytosis | 3 | 6.71 | 1.0E-02 | 3.8E-02 |
| Melanogenesis | 3 | 6.25 | 1.3E-02 | 4.1E-02 |
| Glycine, serine and threonine metabolism | 2 | 13.15 | 1.0E-02 | 3.8E-02 |
| Cysteine and methionine metabolism | 2 | 11.69 | 1.3E-02 | 4.1E-02 |
| *Down-regulated* |  |  |  |  |
| Metabolic pathways | 19 | 3 | 2.4E-05 | 1.2E-03 |
| MAPK signaling pathway | 8 | 5.32 | 1.0E-04 | 2.6E-03 |
| Endocytosis | 5 | 4.43 | 5.7E-03 | 3.6E-02 |
| Glycerophospholipid metabolism | 4 | 8.91 | 1.1E-03 | 1.9E-02 |
| Pancreatic secretion | 4 | 7.06 | 2.6E-03 | 2.7E-02 |
| Vascular smooth muscle contraction | 4 | 6.15 | 4.2E-03 | 3.6E-02 |
| Fat digestion and absorption | 3 | 11.62 | 2.2E-03 | 2.7E-02 |
| Long-term depression | 3 | 7.64 | 7.2E-03 | 3.7E-02 |
| Bacterial invasion of epithelial cells | 3 | 7.64 | 7.2E-03 | 3.7E-02 |
| Ribosome biogenesis in eukaryotes | 3 | 6.68 | 1.0E-02 | 4.1E-02 |
| Fc epsilon RI signaling pathway | 3 | 6.77 | 1.0E-02 | 4.1E-02 |
| VEGF signaling pathway | 3 | 7.03 | 9.1E-03 | 4.1E-02 |
| alpha-Linolenic acid metabolism | 2 | 17.82 | 5.6E-03 | 3.6E-02 |
| Linoleic acid metabolism | 2 | 11.88 | 1.2E-02 | 4.5E-02 |
| Glycine, serine and threonine metabolism | 2 | 11.14 | 1.4E-02 | 4.7E-02 |

^a^Enrichment relative to number of reference genes in the genome based on the hypergeometric test. ^b^Hypergeometric test based. ^c^Benjamini & Hochberg multiple test correction.

**Supp. Table S8. IPA top up-regulated biological functions and canonical pathways**

| Top Biological Functions | | *P* value^a^ | No. of Molecules |
| --- | --- | --- | --- |
|  | |  |  |
| Molecular and Cellular Functions | |  |  |
|  | Cellular Movement | 2.97E-05 - 4.79E-02 | 28 |
|  | Cell Morphology | 6.27E-04 - 4.07E-02 | 17 |
|  | Cellular Assembly and Organization | 6.27E-04 - 4.07E-02 | 17 |
|  | Cell Function and Maintenance | 6.27E-04 - 4.29E-02 | 27 |
|  | Cell Death and Survival | 6.60E-04 - 4.07E-02 | 13 |
|  | |  |  |
| Physiological System Development and Function | |  |  |
|  | Connective Tissue Development and Function | 6.27E-04 - 3.07E-02 | 4 |
|  | Hematological System Development and Function | 1.08E-03 - 4.98E-02 | 19 |
|  | Immune Cell Trafficking | 1.08E-03 - 4.79E-02 | 10 |
|  | Embryonic Development | 1.64E-03 - 4.79E-02 | 8 |
|  | Hair and Skin Development and Function | 1.64E-03 - 2.05E-02 | 8 |
|  | |  |  |
| Top Canonical Pathways | | **-Log (P-value)**^b^ | **Ratio**^c^ |
|  | PI3K Signaling in B Lymphocytes | 1.73E-03 | 0.04 |
|  | Hepatic Fibrosis / Hepatic Stellate Cell Activation | 2.98E-03 | 0.04 |
|  | Glycine Betaine Degradation | 4.51E-03 | 0.20 |
|  | Role of NANOG in Mammalian Embryonic Stem Cell Pluripotency | 5.43E-03 | 0.04 |
|  | Neuroprotective Role of THOP1 in Alzheimer's Disease | 8.06E-03 | 0.07 |
|  |  |  |  |
| ID | **Associated Network Functions** |  | **Score**^d^ |
| 1 | Hematological System Development and Function, Tissue Morphology, Cancer |  | 43 |
| 2 | Cellular Movement, Hematological System Development and Function, Immune Cell Trafficking |  | 21 |
| 3 | Cellular Movement, Lipid Metabolism, Small Molecule Biochemistry |  | 17 |
| 4 | Cell Cycle, Organismal Development, Cancer |  | 15 |

^a^Fischer's exact test was used to calculate a p-value determining the probability that each biological function assigned to that data set is due to chance alone. ^b^Fischer's exact test was used to calculate a p-value determining the probability that each canonical pathway assigned to that data set is due to chance alone. ^c^Number of genes in a pathway that were found in our significant gene list compared to the total number of genes in that pathway. ^d^Network score is the negative log of the p-value for the likelihood that network molecules would be found together by chance alone. A higher score indicates a greater statistical significance that molecules depicted in the network are interconnected.

**Supp. Table S9. IPA top down-regulated biological functions and canonical pathways**

| Top Biological Functions | | *p* value^a^ | No. of Molecules |
| --- | --- | --- | --- |
|  | |  |  |
| Molecular and Cellular Functions | |  |  |
|  | Carbohydrate Metabolism | 3.53E-03 - 4.53E-02 | 6 |
|  | Cell Morphology | 3.53E-03 - 4.53E-02 | 6 |
|  | Lipid Metabolism | 3.53E-03 - 4.65E-02 | 13 |
|  | Molecular Transport | 3.53E-03 - 4.65E-02 | 8 |
|  | Small Molecule Biochemistry | 3.53E-03 - 4.65E-02 | 16 |
|  | |  |  |
| Physiological System Development and Function | |  |  |
|  | Reproductive System Development and Function | 3.53E-03 - 4.53E-02 | 6 |
|  | Connective Tissue Development and Function | 8.06E-03 - 4.53E-02 | 8 |
|  | Cardiovascular System Development and Function | 8.50E-03 - 3.41E-02 | 8 |
|  | Organismal Development | 8.50E-03 - 4.53E-02 | 12 |
|  | Tissue Development | 8.50E-03 - 4.53E-02 | 12 |
|  | |  |  |
| Top Canonical Pathways | | **-Log (p-value)**^b^ | **Ratio**^c^ |
|  | L-DOPA Degradation | 2.29E-02 | 0.50 |
|  | Glutamine Degradation I | 2.29E-02 | 0.50 |
|  | Dopamine Degradation | 2.4E-02 | 0.08 |
|  | Phospholipases | 2.54E-02 | 0.05 |
|  | 1,25-dihydroxyvitamin D3 Biosynthesis | 3.41E-02 | 0.33 |
|  |  |  |  |
| ID | **Associated Network Functions** |  | **Score**^d^ |
| 1 | Cardiovascular System Development and Function, Tissue Development, Cellular Development |  | 42 |
| 2 | Cell-To-Cell Signaling and Interaction, Tissue Development, RNA Post-Transcriptional Modification |  | 19 |
| 3 | Organismal Development, Cellular Development, Cellular Growth and Proliferation |  | 19 |
| 4 | Cellular Development, Cellular Growth and Proliferation, Cell Cycle |  | 17 |

^a^Fischer's exact test was used to calculate a *p*-value determining the probability that each biological function assigned to that data set is due to chance alone. ^b^Fischer's exact test was used to calculate a *p*-value determining the probability that each canonical pathway assigned to that data set is due to chance alone. ^c^Number of genes in a pathway that were found in our significant gene list compared to the total number of genes in that pathway. ^d^Network score is the negative log of the *p*-value for the likelihood that network molecules would be found together by chance alone. A higher score indicates a greater statistical significance that molecules depicted in the network are interconnected.

**Supp. Table S10. Cell cycle analysis with propidium iodide**

| Phase | Control (%) | c.71+9C>A (%) | *p-*value*^a^* |
| --- | --- | --- | --- |
| Dip G1 | 60.42 ± 2.88 | 66.72 ± 2.71 | 0.13 |
| Dip G2 | 14.99 ± 3.29 | 6.79 ± 0.78 | 0.03 |
| Dip S | 24.53 ± 1.42 | 26.49 ± 2.14 | 0.46 |
| Apoptosis | 3.80 ± 1.54 | 18.35 ± 5.84 | 0.05 |

**^a^***p*-value of *t*-test statistic
